# The epigenetically regulated miR-494 associates with stem-cell phenotype and induces sorafenib resistance in hepatocellular carcinoma

**DOI:** 10.1038/s41419-017-0076-6

**Published:** 2018-01-05

**Authors:** Daniela Pollutri, Clarissa Patrizi, Sara Marinelli, Catia Giovannini, Elena Trombetta, Ferdinando A. Giannone, Maurizio Baldassarre, Santina Quarta, Y. P. Vandewynckel, A. Vandierendonck, H. Van Vlierberghe, Laura Porretti, Massimo Negrini, Luigi Bolondi, Laura Gramantieri, Francesca Fornari

**Affiliations:** 1 Center for Applied Biomedical Research, St. Orsola-Malpighi University Hospital, 40138 Bologna, Italy; 2 Center for Regenerative Medicine, Department of Biomedical Sciences, Modena and Reggio Emilia University, 41125 Modena, Italy; 3 Department of Medical and Surgical Sciences, Bologna University, 40138 Bologna, Italy; 4 Flow Cytometry Service, Fondazione IRCCS Ca’ Granda Ospedale Maggiore Policlinico, 20122 Milan, Italy; 5 Department of Medicine, Padua University, 35128 Padua, Italy; 6 Department of Hepatology and Gastroenterology, Ghent University, 9000 Ghent, Belgium; 7 Department of Morphology, Surgery and Experimental Medicine, University of Ferrara, 44100 Ferrara, Italy

## Abstract

Hepatocellular carcinoma (HCC) represents the second cause of cancer-related mortality worldwide and is associated with poor prognosis, especially in patients not amenable for curative treatments. The multi-kinase inhibitor sorafenib represents the first-line treatment option for advanced HCC; nevertheless, its effectiveness is limited due to tumor heterogeneity as well as innate or acquired drug resistance, raising the need for new therapeutic strategies. MicroRNAs (miRNAs) involvement in treatment response as well as their safety and efficacy in preclinical models and clinical trials have been widely documented in the oncologic field, including HCC. Here, we identified miR-494 upregulation in a subgroup of human and rat HCCs with stem cell-like characteristics, as well as multiple epigenetic mechanisms involved in its aberrant expression in HCC cell lines and patients. Moreover, we identified p27, puma and pten among miR-494 targets, contributing to speed up cell cycle progression, enhance survival potential in stressful conditions and increase invasive and clonogenic capabilities. MiR-494 overexpression increased sorafenib resistance via mTOR pathway activation in HCC cell lines and, in line, high miR-494 levels associated with decreased sorafenib response in two HCC animal models. A sorafenib-combined anti-miR-494-based strategy revealed an enhanced anti-tumor potential with respect to sorafenib-only treatment in our HCC rat model. In conclusion, our findings suggested miR-494 as a possible therapeutic target as well as a candidate biomarker for patient stratification in advanced HCC.

## Introduction

Hepatocellular carcinoma (HCC) is the second leading cause of cancer-related mortality worldwide accounting for 90% of primary liver cancers. HCC prognosis is very poor in patients not amenable of curative treatments, with a median survival of less than one year^1^ and an overall ratio of mortality to incidence of 0.95 (http://globocan.iarc.fr/). The lethality of advanced liver cancer is to ascribe to the suboptimal effectiveness of systemic treatments as well as the lack of treatment response biomarkers. At present, the only approved first-line drug for advanced HCC is the multi-kinase inhibitor sorafenib, which improves overall survival of three months^2^ in the presence of relevant adverse events. The high molecular heterogeneity of HCC contributes to compromise the effectiveness of targeted therapies^3,4^. Thus, the identification of innovative therapeutic strategies remains an unmet clinical need in HCC.

Several studies reported the involvement of microRNA deregulation in HCC pathogenesis and drug resistance^5–9^ and, since the liver is easily accessible to systemic gene therapy, miRNA-based strategies have been proposed as potential therapeutic approaches in HCC models and clinical trials^10–15^. MiR-494 belongs to the widest miRNA cluster located in DLK1-DIO3 imprinted locus, which upregulation is found in a stem-like HCC subgroup with poor prognosis and is responsible, itself, for liver cancer development in mice^16–18^. MiR-494 overexpression increased cell cycle progression and promoted cell invasion and migration by targeting *MCC* and *PTEN*, whereas its inhibition decreased nodule size of MYC-driven mice liver tumors^18,19^. In xenografts, miR-494-mediated pten inhibition activated the PI3K/Akt oncogenic pathway favoring the accumulation of tumor-expanded myeloid-derived suppressor cells in tumor microenvironment and facilitating metastatic tumor spreading^20^. Moreover, ERK1/2^−^-dependent activation of miR-494 in non-small cell lung cancer induced tumor resistance to TRAIL treatment through *BIM* targeting^21^.

Here, we investigated the association between miR-494 expression and stem cell characteristics in preclinical models and HCC patients. We also analyzed the multi-target activity of miR-494 as well as its complex epigenetic regulation and demonstrated miR-494-associated mTOR pathway activation as a sorafenib resistance mechanism in HCC.

## Results

### MiR-494 is overexpressed in a HCC subgroup and correlates with tumor size and stemness markers in preclinical models

**Fig. 1:**
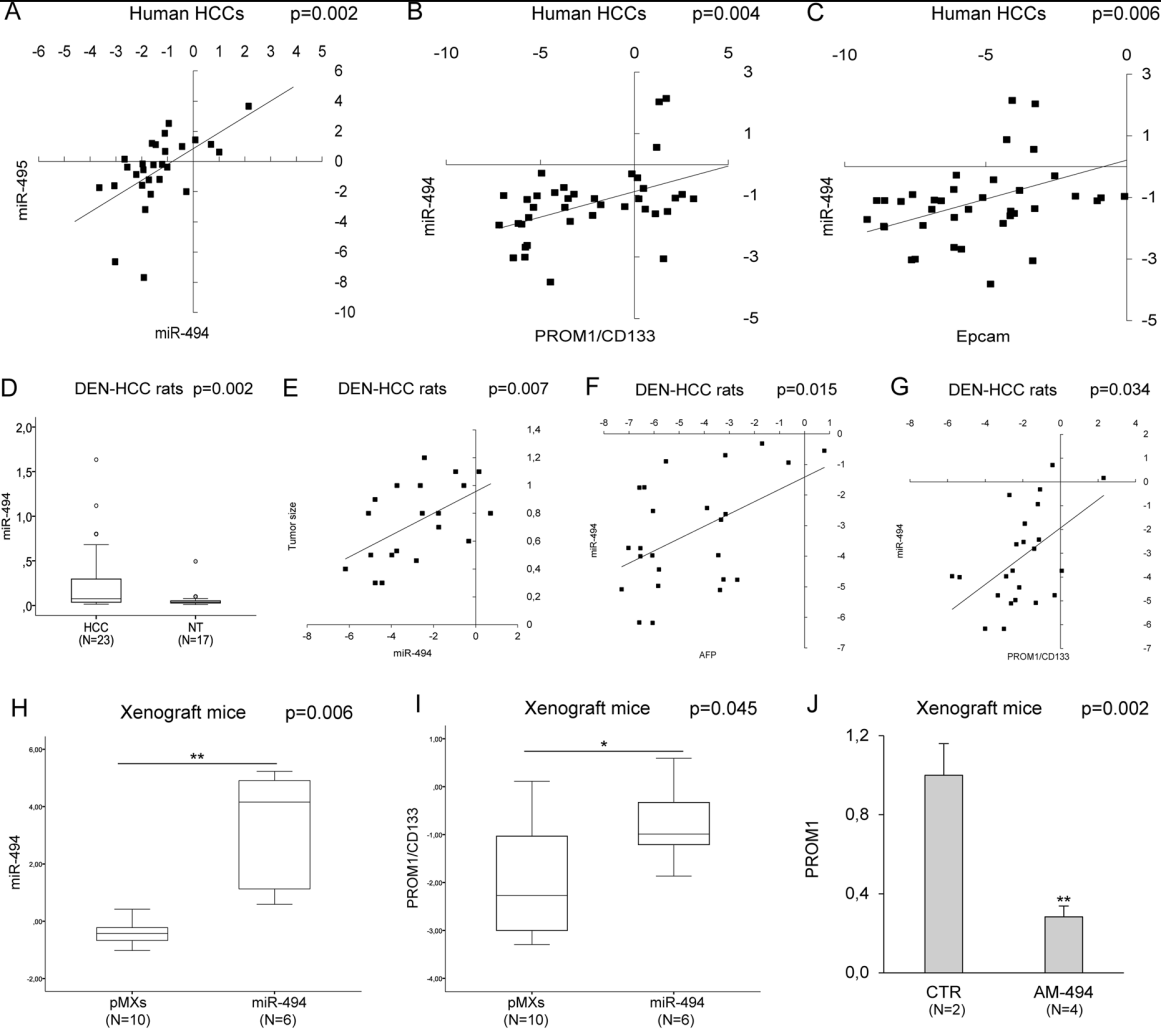
MiR-494 is overexpressed in HCC and correlates with stem cell markers **a** Correlation graph between miR-494 and miR-495 expression levels in tumor tissue from 28 randomly selected HCC patients. Axes report 2^−ΔΔCt^ values corresponding to miRNA levels (log2 form). **b** Correlation graph between miR-494 and *PROM1* or **c** *EPCAM* mRNA levels in tumor samples from 38 HCC patients. Axes report 2^−ΔΔCt^ values corresponding to miRNA and mRNA levels (log2 form). **d** Box plot graph of miR-494 expression in tumor (HCC) and non-tumor (NT) samples from the HCC rat model. *y*-axis reports 2^−ΔΔCt^ values corresponding to miR-494 expression. **e** Correlation graph between tumor size and miR-494 levels in HCC animals. *x*-axis reports 2^−ΔΔCt^ values corresponding to miR-494 levels transformed in a log2 form; *y*-axis represents tumor size (cm). **f** Correlation graph between miR-494 and *AFP* or **g** *PROM1* mRNA levels in tumor samples from HCC rats. Axes report 2^−ΔΔCt^ values corresponding to miRNA and mRNA levels (log2 form). **h** Box plot graph of miR-494 or **i** *PROM1* levels in control (pMXs) and miR-494 overexpressing tumor masses from xenograft mice. *y*-axes report 2^−ΔΔCt^ values corresponding to miR-494 or *PROM1* expression (log2 form). **j** QPCR analysis of miR-494 expression in xenograft mice following antagomiR-494 treatment. CTR: vehicle control mice, AM-494: anti-miR-494 injected mice. *y*-axis reports 2^−ΔΔCt^ values corresponding to miR-494 levels. **a–j** U6RNA and β-actin were used as housekeeping genes

Our previous data reported an aberrant expression of circulating miR-494 in cirrhotic patients with HCC and a positive correlation between serum and tissue levels^22^; therefore, we wondered if miR-494 deregulation might represent a key event in hepatocarcinogenesis (Supplementary Fig. S1). We investigated miR-494 expression in tumors and surrounding livers from 75 surgically resected HCC patients, showing a 2.4-fold upregulation of miR-494 in 25% of tumors compared to matched cirrhosis. Since miR-494 and miR-495 were shown to be the most potent cluster members influencing tumor cell proliferation^18^, we also analyzed miR-495 expression in HCCs. A positive correlation between miR-494 and miR-495 was found in tumors (Pearson’s correlation; *p* = 0.002) but not in surrounding livers (Fig. 1a, Supplementary Fig. S2A), suggesting their possible involvement in hepatocytes malignant transformation. MiR-494 expression correlated with stem cell markers *PROM1/CD133* and *EPCAM* in HCCs (Pearson’s correlation; *p* = 0.004; *p* = 0.006, respectively) (Fig. 1b, c), but not in cirrhosis, confirming miR-494 aberrant expression and its correlation with stemness markers as cancer-specific events^16^. A positive correlation between *PROM1* and *EPCAM* mRNAs was found in tumor and non-tumor tissues (Pearson’s correlation; *p* < 0.0001) (Supplementary Fig. S2B, C), whereas no correlation between miR-494 and other stem-associated genes (*AFP*, *NESTIN*, *CD90*, and *ABCG2*) was found in HCCs.

To study miR-494 role in vivo, we assayed miR-494 expression in DEN-HCC rats mirroring human disease complexity^23,24^. Higher miR-494 levels were detected in 83% of HCCs with respect to non-tumor samples with a 4.6-folds increase (*t*-test; *p* = 0.002) (Fig. 1d). MiR-494 correlated with tumor size (Pearson’s correlation; *p* = 0.007) as well as with *AFP*, *PROM1*, and *ABCG2* expression (Pearson’s correlation; *p* = 0.015, *p* = 0.034, and *p* = 0.023, respectively) (Fig. 1e–g, Supplementary Fig.  S2D); on the contrary, no correlation with *EPCAM* mRNA was found. MiR-494 association with stemness features was confirmed also at a protein level in human and rat HCCs (Supplementart Fig. S2E, F).

A xenograft model was considered to investigate miR-494 involvement in tumor growth. QPCR analysis verified miR-494 overexpression in pMXs-miR-494 Huh-7 cells (Supplementary Fig. S2G) and in tumors derived from this cell clone in comparison with control cells (*t*-test; *p* = 0.006) (Fig. 1h). Strikingly, the fold-change between miR-494-overexpressing and control cells was higher in vivo than in vitro (22.5 vs. 6.7-folds, respectively) (Fig. 1h, Supplementary Fig. S2G), letting us to speculate that a possible crosstalk between tumor and stroma cells might contribute to miR-494 expression. Any difference in tumor size, doubling time, and Ki67 staining was observed when comparing miR-494 with control Huh-7-derived masses, suggesting that higher miR-494 levels do not influence tumor attachment and proliferation in our xenograft model. Nevertheless, as observed in human and rat HCCs, higher *PROM1* levels were displayed in miR-494-derived tumors (*t*-test; *p* = 0.045) (Fig. 1i, Supplementary Fig. S2H). MiR-494 in vivo silencing decreased *PROM1* expression in miRNA-overexpressing xenografts (*t*-test; *p* = 0.002) (Fig. 1j), suggesting miR-494 influence on *PROM1*-specific regulation. These data showed the involvement of miR-494 in HCC pathogenesis as well as in stem cell phenotype of liver tumors.

### MiR-494 is epigenetically regulated in HCC

**Fig. 2:**
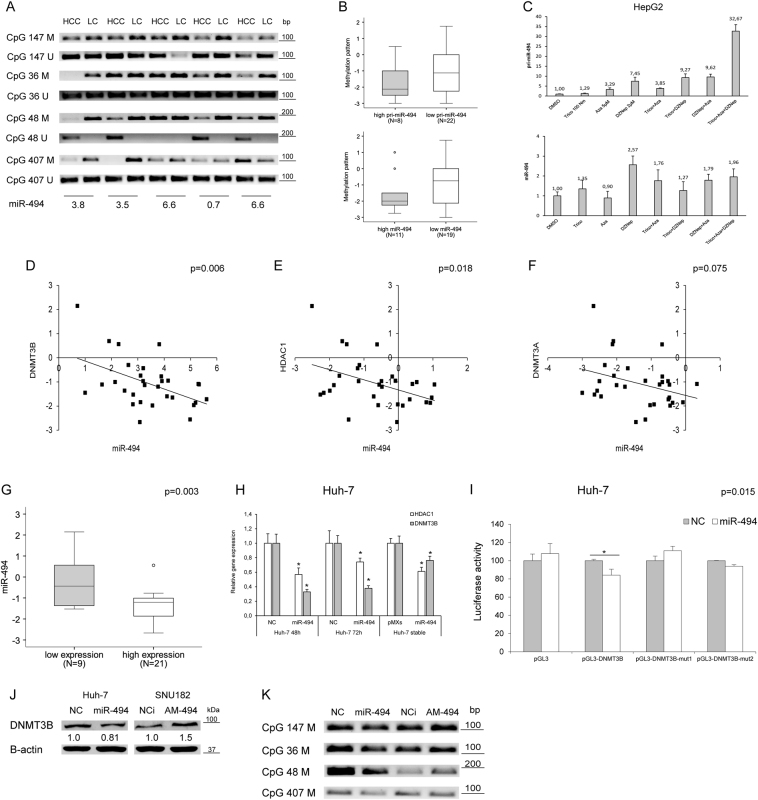
Epigenetic regulation of miR-494 expression in HCC **a** MSP analysis of four CpG islands in tumor (HCC) and liver cirrhotic (LC) samples from 30 HCC patients. Primers for both methylated (M) and unmethylated (U) DNA regions have been used for each CpG island. MiR-494 levels are represented as the ratio between HCC and LC tissue. **b** Box plot graph of methylation status in HCC patients with high or low primary or mature miR-494 expression levels in tumor tissues with respect to matched non-tumor samples. A 1.3-fold-change has been considered as a cutoff to discriminate between high or low primary and mature miR-494 expression levels in HCC vs. matched LC tissues. A qualitative score was assigned to each CpG island based on its methylation status in the tumor vs. non-tumor sample. A mean value of the four CpG regions was considered for each patient. *y*-axis reports the methylation pattern, where negative and positive values are representative of a hypomethylated and hypermethylated status, respectively. **c** QPCR of primary (pri-miR-494) or mature miR-494 levels in HepG2 cells following epigenetic treatments. *y*-axis reports relative miR-494 or pri-miR-494 expression values with respect to vehicle (DMSO)-treated samples. **d** Correlation graphs between miR-494 and *DNMT3B* or **e** *HDAC1* or **f** *DNMT3A* mRNAs in HCCs (*N* = 30). Axes report 2^-ΔΔCt^ values corresponding to miRNA or mRNA levels (log2 form). **g** Box plot graph of miR-494 expression in HCC tumors divided on the basis of high or low *HDAC1* and *DNMT3B* expression with respect to their median values. In particular, ‘‘low expression’’ includes samples with contemporaneous low *HDAC1* and *DNMT3B* levels, whereas ‘‘high expression’’ groups all the other samples. *y*-axis reports 2^-ΔΔCt^ values corresponding to miR-494 levels (log2 form). **h** QPCR analysis of *HDAC1* or *DNMT3B* expression in transfected or infected Huh-7 cells. *y*-axis reports relative miR-494 values with respect to negative controls (NC or pMXs). **i** Luciferase reporter assay in Huh-7 cells co-transfected with pGL3-DNMT3B wild-type or mutated (mu1 and mut2) vectors and miR-494 or negative control (NC). **j** WB analysis of dnmt3b in miR-494 overexpressing or silenced HCC cells. β-actin was used to normalize QPCR and WB data. NC: pre-miR negative control, NCi: anti-miR negative control, AM-494: anti-miR-494. **k** MSP analysis of the four tested CpG islands in miR-494 overexpressing and silenced (AM-494) HepG2 cells. NC: pre-miR negative control, NCi: anti-miR negative control

To have an insight on miR-494 regulation, a methylation-specific PCR (MSP) analysis of selected CpG islands (Supplementary Fig. S2I) was conducted in HCC patients. A hypomethylation pattern was observed in 60% of tumors with respect to surrounding livers (Fig. 2a) in the absence of any association with primary and mature miR-494 levels (Fig. 2b), letting us to hypothesize DNA demethylation as a not a sufficient condition for miR-494 overexpression. CpG48 demethylation was detected only in tumors, suggesting its occurrence as a peculiar cancer-associated event.

To investigate if multiple epigenetic events might be involved in miR-494 regulation, HepG2 cells were treated with 5-Aza-2’-deoxycitidine (5-Aza), Trichostatin (TRC) and 3-Deazaneplanocin A (DZNep), inhibiting DNA methyl-transferases, histone deacetylases, and methyl-transferases. An upregulation of pri-miR-494 was displayed in presence of epigenetic agents, with a stronger effect of DZNep-combined treatments (Fig. 2c). Mature miR-494 levels only partially mirrored pri-miRNA levels, letting us to speculate that other mechanisms might be responsible for its maturation process. In line, a positive but not strong correlation between primary and mature miR-494 was observed in HCC patients and cells (Supplementary Fig. S2J, K).

At the light of our findings and because of incomplete data regarding epigenetic regulation of DLK1-DIO3 miRNAs in HCC^17^, we investigated epigenetic auto-regulatory loops contributing to miR-494 expression. To this aim, a qPCR analysis of chromatin regulating genes was performed in HCC patients. A negative correlation between miR-494 and *DNMT3B* or *HDAC1* mRNAs was observed in tumors (Pearson’s correlations; *p* = 0.006 and *p* = 0.018) (Fig. 2d, e), whereas a trend toward a negative correlation was detected with *DNMT3A* (Pearson’s correlation; *p* = 0.075) (Fig. 2f); on the contrary, no correlation was found with *HDAC2*, *HDAC3,* or *HDAC4*. Combination of low *HDAC1* and *DNMT3B* levels strongly associated with higher miR-494 levels (*t*-test; *p* = 0.003) (Fig. 2g) and, consistently, a decrease of *HDAC1* and *DNMT3B* mRNAs was detected in miR-494-overexpressing cells (Fig. 2h). *DNMT3B* and *DNMT3A* are miR-494 hypothetical targets (Supplementary Fig. S2L), whereas *HDAC1* does not display complementar-binding sites. Since *DNMT3B* showed three binding sites and the highest inverse correlation with miR-494, we verified miR-494/*DNMT3B* mRNA interaction by performing a reporter assay. The luciferase activity of wild-type DNMT3B-3'UTR-vector decreased in miR-494 co-transfected cells in comparison to control cells (*t*-test; *p* = 0.015) (Fig. 2i). To ascertain miR/mRNA interaction, we mutated two miR-494 seed sequences exhibiting the highest likelihood of mRNA downregulation (Supplementary Fig. S2L). Any decrease of luciferase signal was detected for both mutated vectors in miR-494-overexpressing cells (Fig. 2i). Western blot analysis showed a downregulation of dnmt3b in miR-494-overexpressing Huh-7 cells and an upregulation in anti-miR-494-transfected SNU182 cells (Fig. 2j), chosen based on miR-494 basal levels (Supplementary Fig. S3A), demonstrating *DNMT3B* as a miR-494 direct target in HCC. To verify if *DNMT3B* regulation by miR-494 itself might be responsible for CpG island hypomethylation, a MSP analysis was performed in transfected HepG2 cells. A demethylation pattern was observed in miR-494-overexpressing cells, whereas a hypermethylation status was detected in miR-494-silenced cells, with CpG48 displaying the most significant variation (Fig. 2k). These findings demonstrated that an intricate network of epigenetic events regulate miR-494 transcription and that, in turn, it establishes complex feedback loops, by inhibiting *DNMT3B* and *HDAC1* expression in HCC.

### MiR-494 targets p27, pten, and puma in HCC

**Fig. 3:**
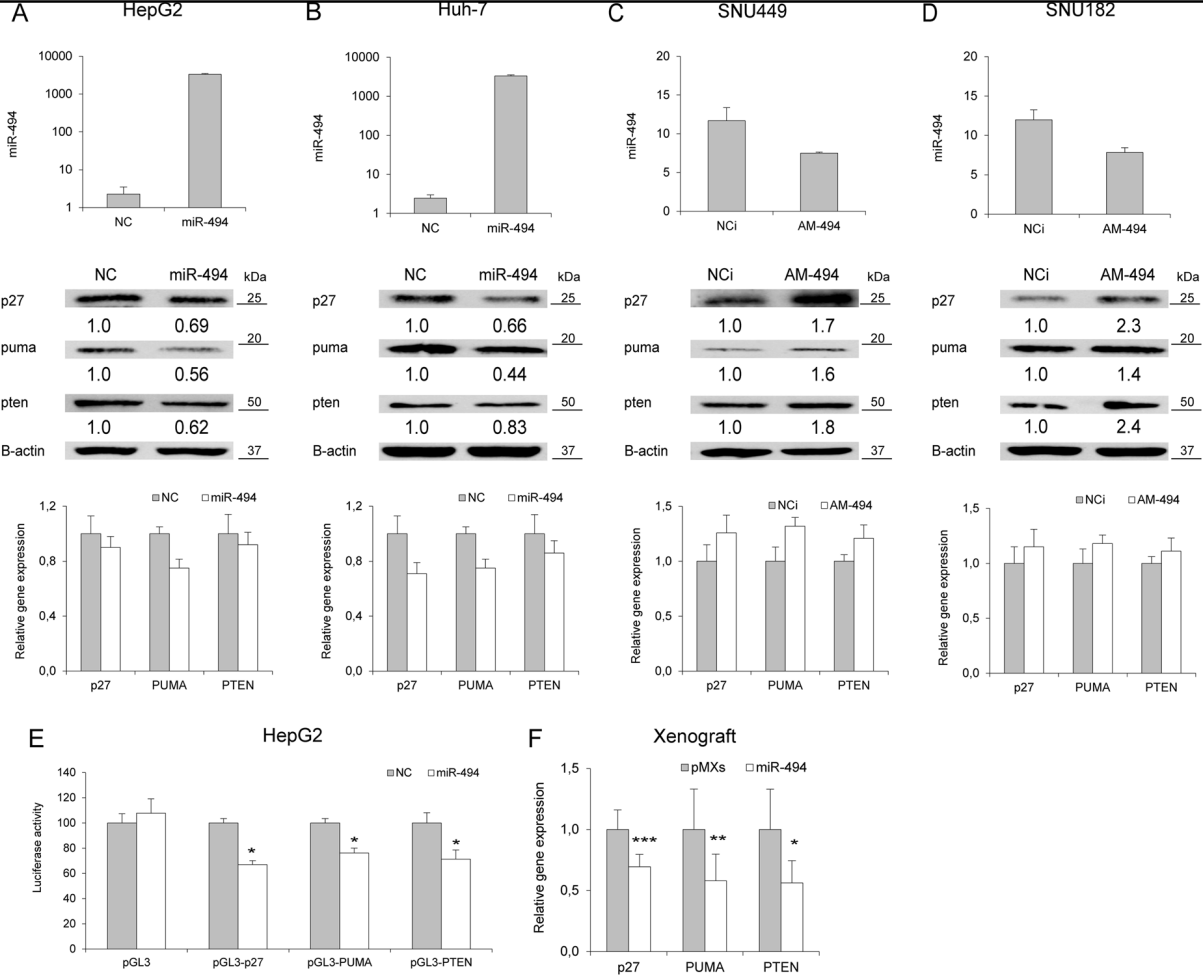
MiR-494 targets *CDKN2B*, *BBC3* and *PTEN* in HCC **a** QPCR and WB analyses of miR-494 and target genes in miR-494 overexpressing HepG2 and **b** Huh-7 cells. *y*-axes report 2^-ΔΔCt^ values corresponding to miR-494 levels (top graphs) or relative gene expression levels (bottom graphs). **c** QPCR and WB analyses of miR-494 and target genes in miR-494 silenced SNU449, and **d** SNU182 cells. *y*-axes report 2^-ΔΔCt^ values corresponding to miR-494 levels (top graphs) or relative gene expression levels (bottom graphs). **e** Luciferase reporter assay in HepG2 cells co-transfected with pGL3-3UTR vectors and miR-494 or negative control (NC). **f** QPCR analysis of miR-494 targets in the xenograft model. *y*-axis reports relative gene expression values. pGL3: empty reporter vector, NC: pre-miR negative control, NCi: anti-miR negative control, AM-494: anti-miR-494. β-actin was used to normalize qPCR and WB data

Aiming to identify key pathways linked to miR-494 aberrant expression, we performed a computational analysis and focused our attention on *CDKN1B/P27*, *PTEN*, and *BBC3/PUMA* (Supplementary Fig. S3B) due to their known roles in cell cycle progression, proliferation and apoptosis. MiR-494 expression was investigated in HCC-derived cells in order to identify the most appropriate model for functional analysis (Supplementary Fig. S3A). MiR-494 overexpression decreased p27, pten and puma proteins in HepG2 and Huh-7 cells (Fig. 3a, b), whereas its silencing increased their levels in SNU449 and SNU182 cells (Fig. 3c, d). MRNA levels were regulated as well, but at a lower extent and depending on cell context (Fig. 3a–d), letting us to speculate that co-regulatory mechanisms might be responsible for their fine-tuning following miR-494 modulation. A decrease of p27, pten, and puma levels was displayed in miR-494-stably overexpressing cells, suggesting a long lasting inhibition in presence of a small miR-494 increase (Supplementary Fig. S3C). The reporter assay showed a decreased luciferase activity of wild type, but not mutant (Supplementary Fig. S3B, D), 3'UTR-vectors in miR-494 co-transfected with respect to control HepG2 cells (*t*-test; *p* < 0.05) (Fig. 3e), demonstrating *PTEN*, *P27* and *PUMA* as miR-494 direct targets in HCC. Lower levels of these target genes were observed in tumors from miR-494-overexpressing cells with respect to empty vector-derived tumors (*t*-test; *p* = 0.0004, *p* = 0.007, and *p* = 0.02, respectively) (Fig. 3f), further confirming our in vitro data.

### MiR-494 regulates invasion capability, cell cycle progression, and stem cell phenotype in HCC

**Fig. 4:**
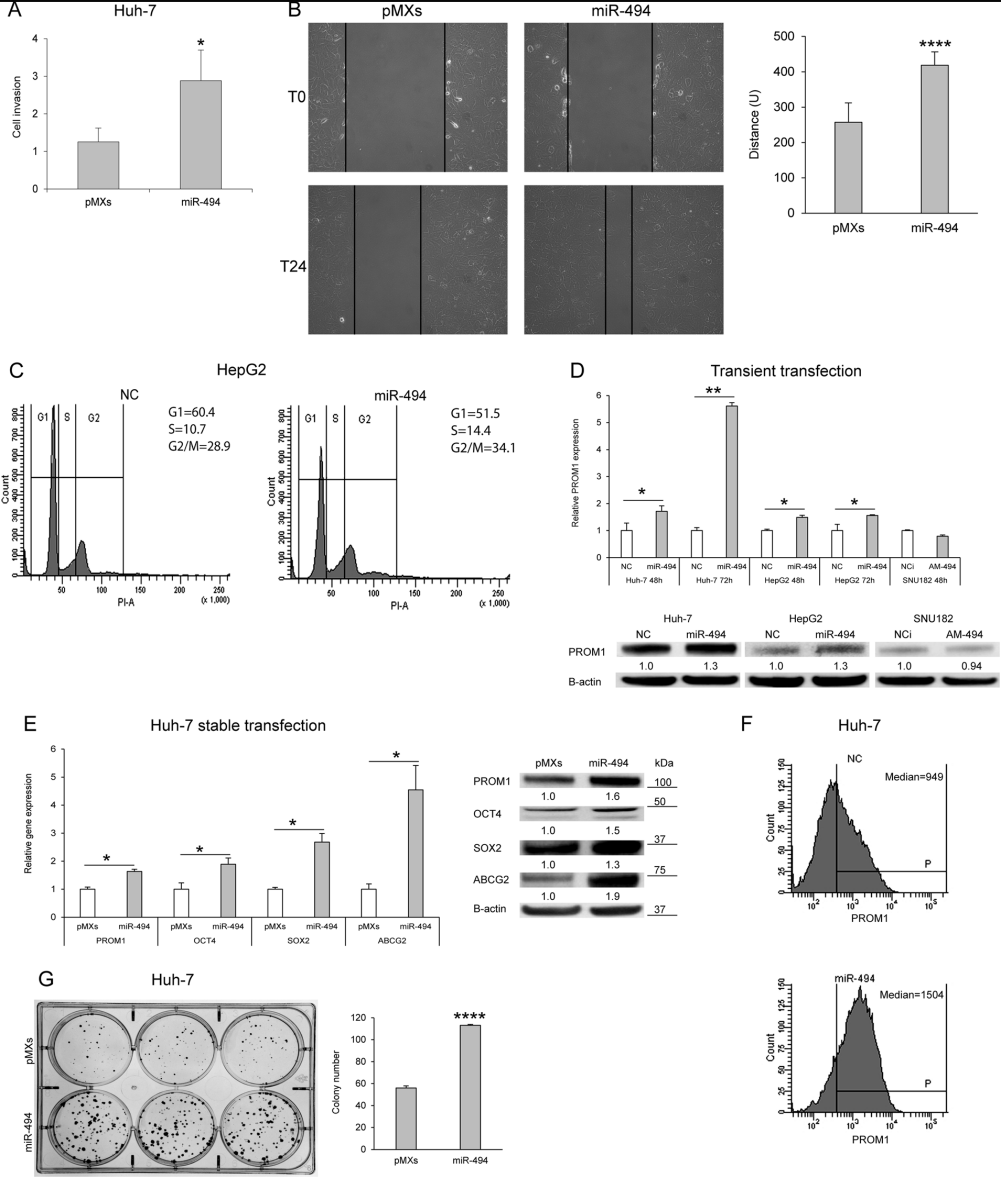
MiR-494 influences cell cycle progression, invasion, and clonogenic capabilities as well as stem cell properties of HCC cells **a** Real-time invasion assay in control (pMXs) and miR-494 infected Huh-7 cells. Mean and SD values of four replicates are reported in the column graph. **b** A wound was made in the monolayer (T0) and the migratory potential of Huh-7 cells was measured following 24 h (T24). Representative images at T0 and T24 and quantification of the wound closure are shown. Columns and bars represent average ± SD values of 10 fields (10X magnification) from two independent experiments. *y*-axis reports arbitrary units (U). **c** Representative cell cycle images of miR-494-transfected HepG2 cells. Cell population percentages are reported on the right top corner of each graph. **d** QPCR and western blot analyses of *PROM1* expression in miR-494 or anti-miR-494-transfected cells. *y*-axis reports relative expression values. NC: pre-miR negative control, NCi: anti-miR negative control, AM-494: anti-miR-494. **e** QPCR and western blot analyses of stem cell markers in infected Huh-7 cells. *y*-axis reports relative expression values. **d**, **e** Experiments were repeated twice in triplicate. **f** FACS analysis of PROM1 immunophenotype in miR-494 overexpressing and negative control (NC) Huh-7 cells. In the right top corner of each histogram is reported the mean value of PROM1 median positivity (P) derived from three independent experiments. **g** Six-well plate image of clonogenic assay and quantification of colony number are shown. All colonies were counted independently from their dimension. Initial number of seeded cells: 250/well. Columns and bars represent average ± SD values of three wells from two independent experiments. **a**, **b**, **e**, **g** PMXs: empty vector infected Huh-7 cells; miR-494: pMXs-miR-494 infected Huh-7 cells

Since *PTEN* plays a pivotal role in cell motility and migration, we assessed invasion and migration capabilities of miR-494-overexpressing Huh-7 cells by using a real-time cell analysis system as well as a wound healing assay. A 2.3-fold increase of invasive potential together with a 1.6-fold enhancement of migration capabilities were observed in miR-494-overexpressing cells (*t*-test; *p* = 0.015 and *p* < 0.0001, respectively) (Fig. 4a, b). Due to the well-established role of p27 as a G1/S checkpoint controller, we tested miR-494 involvement in cell cycle regulation. MiR-494 overexpression in HepG2 and Huh-7 cells displayed a 27% and 23% increase of the S-phase cell population, respectively (*t*-test; *p* = 0.011, and *p* = 0.025) (Fig. 4c and Supplementary Fig. S2E), demonstrating that miR-494 is able to potentiate cell invasiveness and speed up cell cycle progression of HCC cells.

We next assessed miR-494 influence on stemness properties of HCC cells and observed that miR-494 overexpression increased *PROM1*, *OCT4*, and *SOX2* core stemness genes, as well as *ABCG2* transporter levels (Fig. 4d, e). FACS analysis showed a 1.6-fold increase of *PROM1* positivity in miR-494-overexpressing with respect to control cells (*t*-test; *p* < 0.0001) (Fig. 4f). Accordingly, miR-494-overexpressing Huh-7 cells showed a higher clonogenic potential as demonstrated by colony-forming unit assay (*t*-test; *p* < 0.0001) (Fig. 4g and Supplementary Fig. S2F), further supporting miR-494 key role in modulating stem cell phenotype.

### MiR-494 regulates AKT/mTOR pathway and increases cell survival during stress conditions

**Fig. 5:**
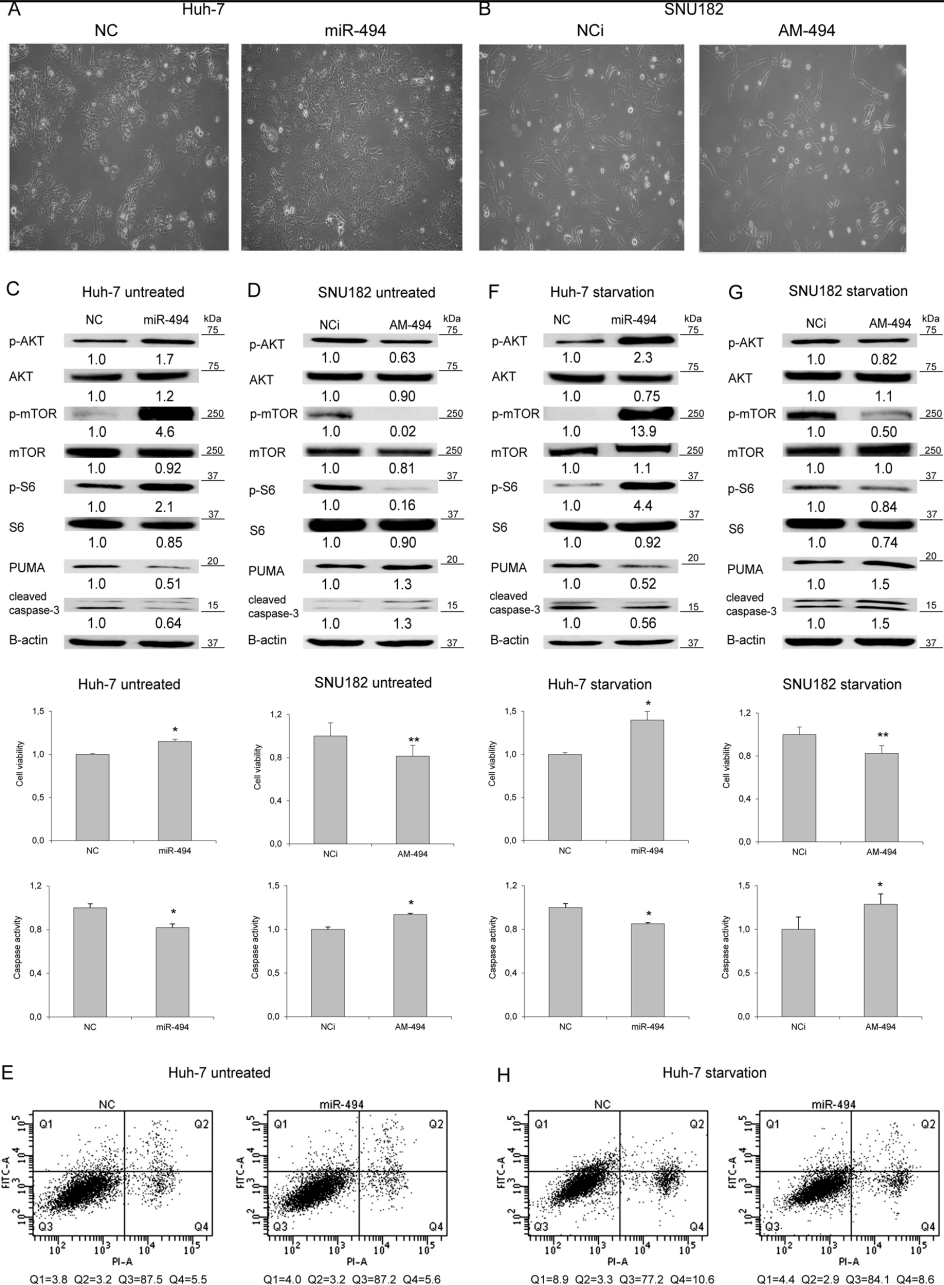
MiR-494 activates the mTOR pathway and increases resistance to stress conditions **a** Cell images of miR-494 overexpressing Huh-7 cells or **b** miR-494 silenced SNU182 cells (10X magnification). **c** Cell viability assay, caspase-3/7 activity assay and WB analysis in untreated miR-494 overexpressing Huh-7 or **d** miR-494 silenced SNU182 cells. **e** FACS Annexin-V plots of miR-494 overexpressing and control untreated Huh-7 (following 48 h of transfection). **f** Cell viability assay, caspase-3/7 activity assay, and WB analysis in miR-494 overexpressing Huh-7 or **g** miR-494 silenced SNU182 cells in starvation condition. **h** FACS Annexin-V plots of miR-494 overexpressing and control Huh-7 following 24 h of starvation. NC: pre-miR negative control. NCi: anti-miR negative control, AM-494: anti-miR-494. β-actin was used to normalize WB data

The most evident phenotypic effect following miR-494 modulation was observed in Huh-7 and SNU182 cells (Fig. 5a, b); therefore, these cell lines were chosen for investigating further miR-494 biological functions. Since pten is the principal negative modulator of Akt/mTOR pathway, we analyzed miR-494 influence on the activation of its downstream signaling cascade. Western blot analysis showed an increase of akt, mtor, and ribosomal S6 phosphorylation levels in miR-494-overexpressing Huh-7 cells (Fig. 5c), whereas miR-494 silencing determined a decrease of their phosphorylation in SNU182 cells (Fig. 5d). No modulation of protein total amount was observed (Fig. 5c, d), suggesting a miR-494-mediated post-transcriptional activation of this pathway.

Due to the well-known role of puma on apoptotic cell death, we performed viability and caspase activity assays in the same settings. An increase of cell viability together with a decrease of caspase activity and cleavage were detected in Huh-7 cells following miR-494 enforced expression (Fig. 5c), whereas an opposite behavior was observed in anti-miR-494-transfected SNU182 cells (Fig. 5d). No variations in cell death were observed in untreated Huh-7 cells in the presence of miR-494 overexpression (Fig. 5e), letting us to speculate that increased viability might be due to a higher proliferation rather than an effective inactivation of apoptosis. Comparable data were obtained in miR-494 stably overexpressing cells (Supplementary Fig. S4A). These findings let us to hypothesize that enhanced miR-494 levels, promoting oncogenic pathway activation and apoptotic signaling inhibition, might protect HCC cells against stressing events commonly observed in the tumor bulk, such as nutrient deprivation and hypoxia. In starvation, an increase of cell viability and akt/mtor phosphorylation, together with decreased apoptotic markers were displayed in miR-494-overexpressing Huh-7 cells (Fig. 5f). In line, miR-494 silencing in serum-deprived SNU182 cells reduced cell viability and increased apoptotic markers (Fig. 5g). A 2.0-fold decrease of early apoptosis was observed in miR-494-overexpressing cells (Fig. 5h), suggesting that miR-494 might strengthen cell resistance to nutrient deprivation by turning off the caspase pathway. In a hypoxia, miR-494 overexpression determined the activation of mTOR pathway, together with an increase of cell viability and a decrease of caspase-3/7 activity in Huh-7 cells. Consistently, higher *HIF1A* levels were observed in miR-494-overexpressing cells in basal and hypoxic conditions (Supplementary Fig. S4B, C), demonstrating the central role for miR-494 in cell survival following stressful events.

### MiR-494 regulates response to treatments in HCC cells

**Fig. 6:**
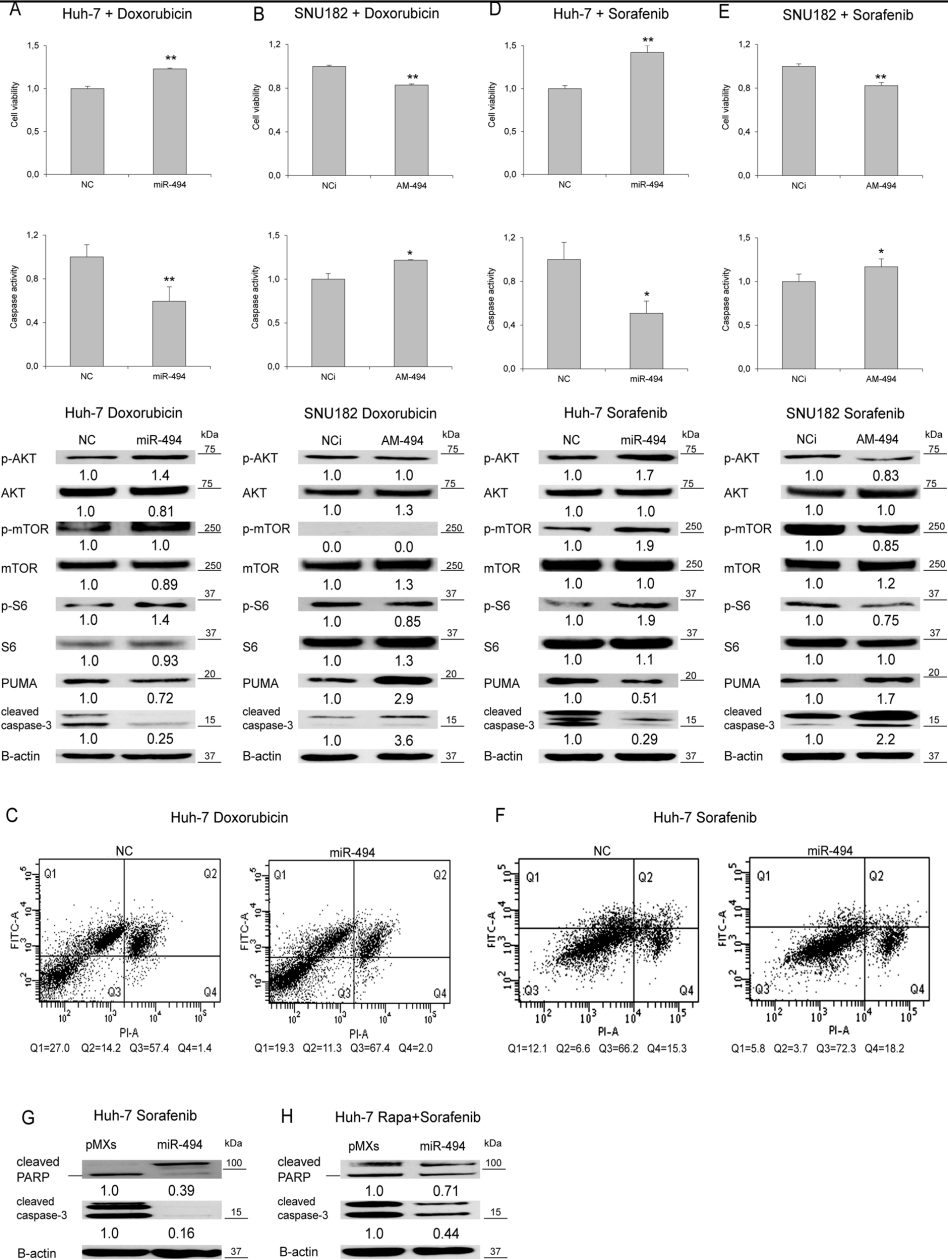
MiR-494 increases drug resistance in HCC cells **a** Cell viability assay, caspase-3/7 activity assay and WB analysis in miR-494 overexpressing Huh-7 or **b** miR-494 silenced SNU182 cells following doxorubicin treatment. **c** FACS Annexin-V plots of miR-494 overexpressing and control Huh-7 cells following doxorubicin treatment (10 µg/ml for 6 h). **d** Cell viability assay, caspase-3/7 activity assay and WB analysis in miR-494 overexpressing Huh-7 or **e** miR-494 silenced SNU182 cells following sorafenib administration. **f** FACS Annexin-V plots of miR-494 overexpressing and control Huh-7 cells following sorafenib treatment (7.5 µM for 24 h). **g** WB analysis of apoptotic markers in miR-494 overexpressing Huh-7 cells following sorafenib or **h** sorafenib plus rapamycin treatment. NC: pre-miR negative control; NCi: anti-miR negative control; AM-494: anti-miR-494. β-actin was used to normalize WB data

To evaluate the role of miR-494 in response to genotoxic damage we employed doxorubicin, a drug used during HCC locoregional treatments. MiR-494-overexpressing Huh-7 cells showed an enhanced resistance to doxorubicin challenge as determined by cell viability and caspase-3/7 assays (Fig. 6a), whereas its downregulation in SNU182 cells increased doxorubicin sensitivity (Fig. 6b), with only a marginal modulation of Akt/mTOR pathway. These data were confirmed by Annexin-V analysis displaying decreased early and late apoptotic events (1.4 and 1.3-folds, respectively) in miR-494-overexpressing cells (*t*-test; *p* < 0.05) (Fig. 6c).

Subsequently, we tested miR-494 biologic effect following sorafenib treatment. MiR-494 overexpression enhanced cell resistance to sorafenib in Huh-7 cells, increasing cell viability, and decreasing caspase activity (Fig. 6d), whereas opposite results were displayed in anti-miR-494-treated SNU182 cells (Fig. 6e). Annexin-V analysis strengthened these data showing a 2.0-fold decrease of early and late apoptosis in miR-494-overexpressing cells after sorafenib administration (*t*-test; *p* < 0.05) (Fig. 6f). A further confirm was obtained in stable miR-494 Huh-7 cells displaying an increased resistance to sorafenib challenge with respect to control cells (Supplementary Fig. S5A). MiR-494-mediated caspase inhibition reflected cell viability and apoptosis variations, suggesting a central role for the caspase cascade in drug resistance of miR-494-overexpressing cells.

High mTOR phosphorylation levels in miR-494-overexpressing cells let us to hypothesize a considerable involvement of this pathway in sorafenib sensitization (Fig. 6d and Supplementary Fig. S2A). To demonstrate this hypothesis, mTOR activity was turned off by using rapamycin^5^. Co-treatment with rapamycin sensitized miR-494-overexpressing cells to sorafenib challenge when compared to sorafenib-only treated cells (Fig. 6g, h), demonstrating a strong participation of the mtor pathway in miR-494-mediated sorafenib resistance, as confirmed by caspase inactivation and decreased PARP levels.

Aiming to rule out off-target effects, miR-494-overexpressing cells were transfected with anti-miR-494 or controls before sorafenib administration (Supplementary Fig. S5B). An increase of caspase activity was detected in anti-miRNA-treated miR-494 Huh-7 cells, resembling the value of empty vector cells (Supplementary Fig. S5C).

### MiR-494 overexpression correlates with sorafenib resistance in HCC animal models

**Fig. 7:**
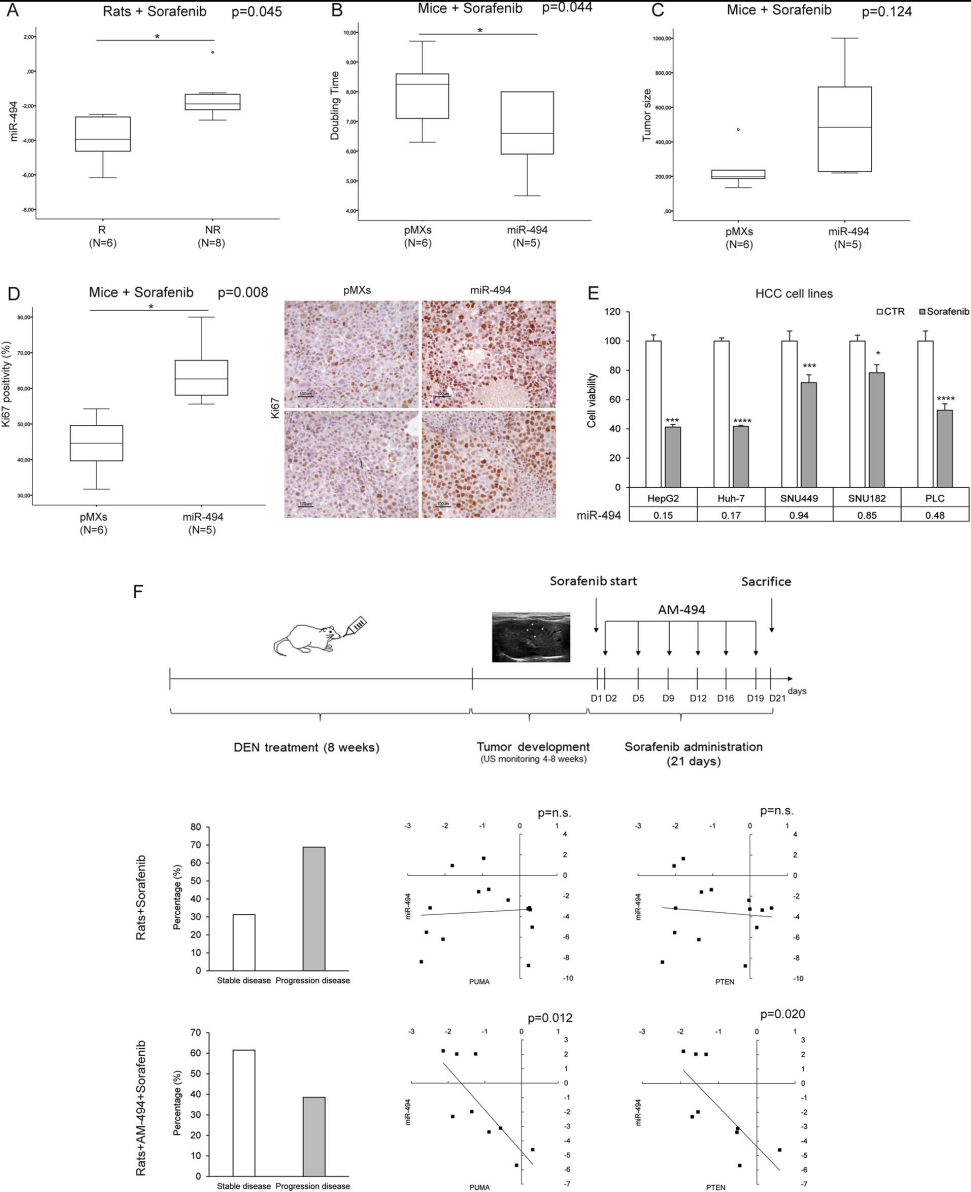
MiR-494 increases sorafenib resistance in vivo **a** Box plot graph of miR-494 expression in responder (R) and non-responder nodules (NR) from Sorafenib treated HCC rats. *y*-axis reports 2^-ΔΔCt^ values corresponding to miR-494 expression (log2 form). **b** Box plot graph of tumor doubling time or **c** size in Huh-7-derived xenografts. *y*-axes report tumor doubling time (days) or size (mm^3^), respectively. **d** Box plot graph of Ki67 positivity in Huh-7-derived xenografts. *y*-axis report tumor cell positivity (%). Representative IHC images (20X magnification) of Ki67 staining in control (pMXs) and miR-494 overexpresing Huh-7-derived xenograft masses. **e** Cell viability assay in HCC cells following sorafenib treatment (5.0 µM for 48 h). Columns represent cell viability relative (%) values with respect to vehicle (CTR)-treated cells. The experiment was executed twice in quadruplicate. MiR-494 basal expression levels (2^−ΔΔCt^) of each cell line are reported in table below the graph. **f** Panel graph of combined Sorafenib and AM-494 in vivo treatment in the DEN orthotopic rat model. In the top part of the panel graph is illustrated the experimental protocol. When US-monitoring identified a 2–3 mm nodule we started with animal treatments, considering as day 1 (D1) the first day of sorafenib administration. Two AM-494 injections were performed weekly starting from the second day (D2). In the bottom part are illustrated percentage graphs representing treatment efficacy together with correlation graphs between miR-494 and PUMA or PTEN mRNA levels in tumor samples from sorafenib-only and anti-miR-494-sorafenib treated animals. Axes report 2^−ΔΔCt^ values corresponding to miRNA and mRNA levels (log2 form). AM-494: anti-miR-494

To investigate the role of miR-494 in sorafenib response in vivo, miR-494 expression was analyzed in HCCs from DEN-treated rats receiving sorafenib intragastrically. Isolated tumors were considered as ‘‘responder’’ and ‘‘non-responder’’ based on US-monitoring and histopathological examination^25^. QPCR analysis displayed an association between high miR-494 levels and sorafenib resistance in rat HCCs (*t*-test; *p* = 0.045) (Fig. 7a). Consistently, the xenograft model showed a lower doubling time (*t*-test; *p* = 0.044) (Fig. 7b) and a trend toward a higher tumor size (*t*-test; *p* = 0.124) (Fig. 7c) in miR-494-overexpressing tumors, suggesting that miR-494 might influence tumor cell proliferation during sorafenib treatment. To verify this hypothesis, Ki67 staining was evaluated displaying an increased positivity (1.5-folds) in miR-494-derived tumors (*t*-test; *p* = 0.008) (Fig. 7d). Cell viability assay displayed an association between high miR-494 basal levels and sorafenib resistance in HCC-derived cells (Fig. 7e), confirming a close relationship linking miR-494 expression to sorafenib response in preclinical models.

The efficacy of a combined miRNA-based strategy was assessed in the rat model following anti-miR-494 and sorafenib co-administration (Fig. 7f). A stabilization of tumor progression was observed in 61% vs. 31% of nodules isolated from anti-miR-494-sorafenib with respect to sorafenib-only treated animals (*χ*^2^ test; *p* < 0.05). A negative correlation between miR-494 and *PUMA* or *PTEN* mRNAs (Pearson’s correlations; *p* = 0.012 and *p* = 0.020, respectively) was found in the combined-treated group but not in the sorafenib-only one (Fig. 7f), suggesting these molecular pathways mediating miR-494 therapeutic effects.

## Discussion

Despite the huge heterogeneity of hepatocellular carcinoma, several profiling studies well-documented the association between deregulated microRNAs and HCC subgroups characterized by defined clinical features as well as molecular and genetic alterations^26–28^. In line with previous findings describing an increase of miR-494 in 34% of tumor tissues and an upregulation of this miRNA cluster in a subclass of HCCs^17,18^, here we detected high miR-494 levels in 25% of tumors and an association with stemness-specific genes. As frequently observed for cancer-associated miRNAs, miR-494 may behave as an oncogene or a tumor-suppressor gene in a tissue-dependent manner. MiR-494 upregulation and involvement in cancer progression was reported in lung, colorectal, and glioblastoma cancers, as well as in HCC^18,19,29,30^. On the contrary, its decreased expression was detected in cholangiocarcinoma, breast, and gastrointestinal stromal tumors^31–33^, letting us to speculate that miR-494 might change its preferential target core depending on tissue context. Here, we showed that miR-494 regulates p27, pten, and puma in HCC cells and xenograft tumors, increasing cell cycle progression, cell survival in stressful conditions and enhancing invasive and clonogenic capabilities. Recently, Lim and coworkers validated *MCC* gene among miR-494 targets in HCC showing its implication in cell cycle transition, as demonstrated by functional analysis and silencing-specific experiments^18^.

The involvement of cancer-related miRNAs in the regulation of treatment response has been extensively documented in HCC^5–7,9,25,34–36^. We observed that miR-494 associated with sorafenib resistance in HCC preclinical models and demonstrated that miR-494-mediated mTOR pathway activation was responsible for decreased targeted therapy sensitization. Consistently, Chen and coworkers demonstrated that PI3K/Akt signaling inhibition is able to restore sorafenib sensitivity in HCC^37^. In agreement, a recent study showed that miR-494-mediated pten regulation is involved in sorafenib resistance through the activation of PI3K/Akt pathway in HepG2 cells^38^. We reported that a combined anti-miR-494-based therapeutic strategy is more efficient in terms of tumor stabilization in comparison to sorafenib-only treatment in DEN-HCC rats. Several studies reported that tumor-promoting miRNAs targeting *PTEN* are involved in drug resistance^9,38–40^ and that their multiple inhibition by a long non-coding RNA-mediated strategy induced sorafenib sensitization in HCC^41^.

Akt/mTOR signaling activation associated with stem cell marker positivity and contributed to the selection of Epcam-positive tumor-initiating cells responsible for sorafenib resistance in HCC^42,43^. Accordingly, we showed the steady association between miR-494 and core stemness genes in preclinical models, as well as in human HCCs, suggesting a key role for miR-494 in *PROM1* transcriptional regulation. A recent paper reported a p53-mediated hdac1 recruitment to *PROM1* promoter causing a decrease of its transcription^44^; since we showed an inverse correlation between miR-494 and *HDAC1* in HCCs, we can speculate that *HDAC1* might participate to *PROM1* regulation in miR-494-overexpressing cells.

Methylation-based profiling of HCC demonstrates the association between epigenetic changes and prognosis, as well as progenitor cell characteristics^45^. However, comprehensive epigenetic profiles considering more events are difficult to be applied and proposed for HCC subgroup characterization. We showed that miR-494 upregulation results from simultaneous epigenetic changes, which is in agreement with previous studies describing the involvement of histone demethylation^46^, but not DNA hypomethylation alone^17^, in enhancing miR-494 expression in cancer cells. Beside epigenetic regulation, our data suggested that post-transcriptional mechanisms might be involved in miRNA biogenesis determining final mature miR-494 levels. Increasing evidences demonstrated the complexity of miRNA processing machinery and reported a tight crosstalk with key intracellular molecules^47–49^, nevertheless further investigations are necessary to unravel the complex network of interactions at the basis of miR-494 deregulation in hepatocarcinogenesis. Regulatory loops involving epigenetic enzymes, such as dnmt3b, hdac1 and tet1, were assessed in HCC, highlighting the complexity of molecular events underlying miR-494 deregulation. Specifically, through the modulation of epigenetic targets, miR-494 is able to remove DNA methylation tags and to trigger gene silencing of invasion-suppressor miRNAs leading to tumor metastasis^50^, as well as to fine-tune its own expression by CpG island demethylation. In this scenario, miR-494 deserves attention as a putative biomarker for the identification of a subgroup of epigenetically distinct HCCs. Notably, our previous findings showed that circulating miR-494 levels correlated with tissue ones in HCC patients^22^, suggesting this miRNA as a non-invasive biomarker. In conclusion, this study illustrates the detrimental effect of miR-494 in sorafenib resistance via mTOR pathway activation and highlights its possible role as a therapeutic target and a candidate biomarker for patient stratification.

## Patients and methods

### Patients

Tumor and cirrhotic tissues were obtained from 75 consecutive patients undergoing liver resection for HCC. Tissues were collected after obtaining an informed consent and were stored as previously described^8^. St. Orsola-Malpighi Hospital approved the study protocol. No patient received anticancer treatment prior to surgery. Patient characteristics are summarized in Table S1.

### HCC animal models

The diethylnitrosamine (DEN)-induced HCC rat model and the xenograft model were established as previously described^25^. The xenograft model was obtained by inoculating miR-494 stably overexpressing (pMXs-miR-494) Huh-7 cells. Anti-miR-494 administration in both models is described in Supplementary Material. At sacrifice, tumor masses were collected for molecular and histopathologic analyses.

### Cell culture and treatments

HCC-derived cell lines were cultured as previously described^25^ and specific treatments are detailed in Supplementary Material. Apoptotic cell death and cell viability were evaluated by Caspase-Glo 3/7 and Cell-titer-Glo assays (Promega, Madison, USA) accordingly to the manufacturer's protocols. Each experiment was performed in quadruplicate. Oligonucleotide transfection of pre-miR-494, anti-miR-494, or negative controls (100 nM, Thermo Fisher Scientific, Whaltam, USA) was obtained by using TransIT-X2 dynamic delivery system (Mirus Bio, Madison, USA) according to the manufacturer's instructions. Cell cycle and Annexin-V analyses were performed in triplicate as previously reported by our group^5,51^. Immunophenotypic analysis of PROM1 expression was performed by using CD133 (Prominin-1) monoclonal antibody (13A4)-APC (eBioscience) diluted 1:5 with respect to the manufacturer's instruction.

### Retroviral infection

DNA sequence of precursor miR-494 was inserted between *Xho*I cloning sites of pMXs-miR-GFP/Puro retroviral expression vector according to the manufacturer's datasheet (Cell Biolabs, San Diego, USA). Primers and PCR conditions are reported in Table S2. Viral infection of Huh-7 cells was performed as previously described^7^.

### Luciferase activity assay

The 3'UTR regions of human *PTEN*, *BBC3*, *CDKN1B*, and *DNMT3B* genes were amplified by PCR using primers and conditions reported in Table S2. The mutagenesis of miR-494 seed sequence in *BBC3*, *CDKN1B*, and *DNMT3B-3'* UTR-containing vectors was performed by using QuikChange II Site-Directed Mutagenesis Kit (Agilent Technologies) following the manufacturer's instruction. Sanger sequencing verified mutated sequences. Oligonucleotide sequences for mutagenesis assay are detailed in Table S2. Luciferase reporter assay of 3'UTR-containing vectors was performed as previously reported^51^.

### Quantitative PCR and semi-quantitative RT-PCR

TaqMan MicroRNA assays (Thermo Fisher Scientific) were used for quantifying miRNA-494 (ID: 002365) and miR-495 (ID:001108) expression, as previously described^8^. RNU6B (ID: 001093) was used as reference gene. Primers and conditions for SYBR-green QPCR and RT-PCR are detailed in Table S3. β-actin was used as housekeeping gene and QPCR experiments were run in triplicate.

### Western blot and immunohistochemistry (IHC)

Thirty micrograms of whole-protein extracts from HCC cells and tissues were used for western blot analysis. Antibodies are reported in Table S4. Digital images of X-ray films were acquired by using ChemiDoc^TM^ XRS + (Image Lab^TM^ Software, Bio-Rad, Hercules, USA). Western blot analysis was performed in triplicate. The IHC of Ki67 (1:800; Agilent Technologies, Santa Clara, USA) in xenograft tumors was assessed on formalin-fixed, paraffin-embedded sections as detailed in Supplementary Material.

### DNA methylation analysis

We analyzed bisulfite-treated DNA samples from HCC patients by MSP as previously described^6^ and as detailed in Supplementary Material. Briefly, one microgram of DNA was treated with bisulfite modification kit (EZ DNA Methylation-Gold kit, Zymo Research) according to the manufacturer's instructions. Modified DNA was eluted in 20 µl of TE buffer and one microliter of modified DNA samples was used for MSP analysis. To evaluate the quality of bisulfite-treated DNA samples, we performed the MSP analysis using unmethylated primers for the same tested CpG islands. The calculation of the methylation pattern is detailed in Supplementary Material and Methods section. Primers and conditions are reported in Table S5.

### Cell invasion and wound healing assay

Real-time analysis of cell invasion was performed on the xCELLigence DP instrument (ACEA, San Diego, USA). Briefly, the surface of the upper chamber wells of a two-chambers device (CIM-plate 16) was coated with a monolayer of 1X collagen I solution (Sigma-Aldrich). Medium with 10% serum was placed in the lower chamber, as chemoattractant. A porous membrane separates the two chambers and cells migrate through a solid matrix at the membrane where the electrodes reside. PMXs and pMXs-miR-494 Huh-7 cells were seeded (25,000 cells/well) in serum free medium in the upper chamber. The cell index (CI, a quantitative measure of cell number present in a well) of each well was measured every 15 min for up to 40 h at 37 °C in 5% CO_2_ atmosphere using the RTC software (version 1.2, Roche Diagnostics) to evaluate the invasion capacity of tested cell lines.

For the wound healing assay, stably overexpressing Huh-7 cells were seeded in a six-well plate (150,000 cells/well) and were grown until monolayer formation. A wound was created in the monolayer using a P200 micropipette tip and cells incubated in complete medium for 24 h. Ten random pictures were taken (10X magnification) when the scratch was performed (T0) and after 24 h (T24). The reduction in the wound gap was determined by using Image-J software (NIH).

### Colony-forming unit assay

Cells were seeded at a low concentration in a six-well plate (250 or 500 cells per well) and incubated in complete medium until colony formation (11 or 9 days, respectively). Cells were washed with PBS, fixed in paraformaldehyde (2% in PBS) for 10 min at room temperature (RT), stained with crystal violet (0.5% in 25% methanol) for 20 min at RT. Digital images were digitally acquired and colony number counted by Image-J software.

### Statistical analysis

Differences between groups were analyzed using unpaired Student’s *t*-test. Pearson’s correlation coefficient was used to explore relationships between two variables. In vitro experiments were performed in triplicate and the mean values were used for the statistical analysis. Reported *p*-values were two-sided and considered significant when lower than 0.05. Statistical calculations were executed using SPSS version 20.0 (SPSS inc). *p < 0.05, **p < 0.01, ***p < 0.001, ****p < 0.0001.

## Electronic supplementary material

Supplemental Material and Figure legends

Supplementary Tables

Figure S1

Figure S2

Figure S3

Figure S4

Figure S5
