# Supplementary material for: The epigenetically regulated miR-494 associates with stem-cell phenotype and induces sorafenib resistance in hepatocellular carcinoma
Source: Cell Death Dis. 2018 Jan 5;9(1):4. doi: 10.1038/s41419-017-0076-6 (PMC5849044; doi:10.1038/s41419-017-0076-6)
Supplement: Supplementary file 1 — Supplemental Material and Figure legends [file 41419_2017_76_MOESM1_ESM.doc]

### Supplementary Material

**HCC rat model**

A further group of HCC animals (N=19) was employed to assess the effect of combined sorafenib and anti-miR-494 (AM-494) therapeutic strategy. Oligonucleotides (10 mg/kg) were suspended in DEPC water and delivered by tail vein injection twice a week for three weeks, together with sorafenib (10 mg/kg) intragastric administration (N=7). The control group (N=12) received only sorafenib (10 mg/kg) for 21 days; animals were euthanized 48 hours after the last intra-venous injection. The sequence of chemically stabilized AM-494 (IDT) is 5’-mA*mC*mUmUmUmGmUmAmUmGmUmGmCmCmCmUmUmU*mG*mG*mA*mG*mA-3’ (where ‘‘m’’ represents 2’-Omethyl RNA bases and asterisk [*] represents phosphothioate bonds). More nodules were harvested for each animal. Local ethics committee approved the protocol (14/70/12).

**Xenograft mouse model**

A pilot study was conducted on further six animals with pMXs-miR-494-derived tumor masses in order to demonstrate the feasibility of anti-miR-494 (AM-494) in vivo delivery. The sequence of chemically stabilized AM-494 (IDT) is 5’-mA*mC*mUmUmUmGmUmAmUmGmUmGmCmCmCmUmUmU*mG*mG*mA*mG-3’ (where ‘‘m’’ represents 2’-Omethyl RNA bases and asterisk [*] represents phosphothioate bonds). AM-494 oligonucleotides were suspended in DEPC water and one single dose (10 mg/kg) administered intraperitoneally (N=4). The control group (N=2) received only the vehicle; animals were euthanized 48 hours later.

# **Cell culture and treatments**

Cells were grown for 48 hours in media without FBS to mimic starvation conditions or at 1% O_2_ in a hypoxic chamber (INVIVO_2_ 300, Baker Ruskinn) to mimic low oxygen conditions. Epigenetic inhibitors were used at the following concentrations for 48 hours: 5-Aza-2’-deoxycitine (Aza) 5 µM; Trichostatin (TRC) 100 nM (Sigma-Aldrich); 3-Deazaneplanocin A (DZnep) 2µM (Selleckchem). Cells were treated with 10 µg/ml of doxorubicin (Pfizer) for 6 hours or 2.5-10.0 µM of sorafenib (Bayer) or 50 ng/ml of rapamycin (Calbiochem) for 48 hours. All experiments were performed in triplicate.

**DNA methylation analysis**

The four tested CpG islands (147, 36, 48, 407), interspersed in the human DLK1-MEG3 locus, were identified by UCSC database (<http://genome.ucsc.edu/>), their DNA sequence is below reported; MSP primers were designed by using MethPrimer Software (<http://www.urogene.org/methprimer/>). A qualitative score, ranging from -3 to +3, was assigned to each CpG island on the basis of the methylation status in the tumor tissue with respect to matched non-tumor sample (e.g.: -3 for highly hypomethylated HCCs; +3 for highly hypermethylated tumors), as shown in the table below reported. The mean score of the four CpG islands was calculated for each patient and this value was considered as representative of his/her methylation pattern in the DLK1-DIO3 region.

**Calculation of methylation score in HCC patients**.

| Representative MSP image in HCC and liver cirrhosis (LC) | 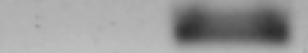 HCC LC | 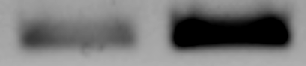HCC LC | 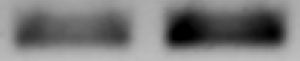HCC LC | 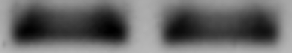HCC LC | 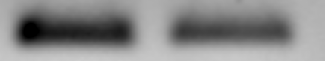HCC LC | 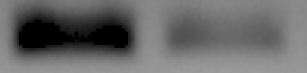HCC LC | 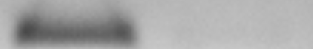HCC LC |
| --- | --- | --- | --- | --- | --- | --- | --- |
| Measured HCC/LC OD ratio | 0.01 | 0.34 | 0.67 | 1.15 | 1.45 | 3.11 | 31 |
| Intervals of HCC/LC OD ratio (R) | ≤0.1 | 0.1<R<0.4 | 0.4≤R≤0.8 | 0.8<R<1.2 | 1.2≤R≤2.5 | 2.5<R<10 | ≥10 |
| Methylation score | -3 | -2 | -1 | 0 | +1 | +2 | +3 |

**DNA sequence of tested CpG islands as determined by UCSC database**

>hg19_cpgIslandExt_**CpG: 147** range=chr14:101004068-101005681 5'pad=0 3'pad=0 strand=+ repeatMasking=none

CGCGTGAAAAACGCTCTAAGTGGAGTTCCACGGGCCGGGGCCGGGTGGAC

ACCGTGGTGTGGGGGACCCTCCCCTCAGGCCTACAGGGCTGGGGAGGAGA

GGAGGTGCGGGGAGGAACAGACTCCTCGTTGTTGGCCGGGGCAGGGGAAC

AGCGGGGGCTGGGGAGAGGTGAGGCCGGCCCTTCTGGGGCACGTGGGCTG

GCGGGGAGCGAACCACGGCCAGGCCTGCACGCAGGCGCTCAGTTGAGCAA

GGTTCCGTAGAGCTGGGCCTTGGTGAGGCTGTCCTTGCGGCTCAGCCCCG

AGCCACCAGTCCGCGGAAAGGCCTGCTGGGGGCTGAGGCGGGCGGCAGGA

TGCATTTCCGGGGAGGCCTCCATGGAGCTCGGCTCCAAGGAGTCCCTGGA

ACCCTGCTCGGGCTCAGGGCTGCTGGCGGTCCCACACAGCTGCACCCCGA

GCCTGTCCCCGTCCCCCCCCTCGCTGGGTGCATAGCCGGGCAGTGGGTCA

GCCGAGCGGCCGGGACTGAGGCTCAGGTCGCCGCCCGCCCGCAGGAAGCA

GGGCTCTGCCAGGTGGCCCTGGGAGAGGTCGTCCCCCTCGGAGAAGCTGT

CGGCCTTGTAGCTGGCGTAGAGCGGGCTGGCGCGGCCGTCGGCCTTCTTG

CCCGGGCTGCCCCCGGCCCCGCCGTAGTAGCGTTCAGAGAAGCTGCAGGG

TGAGGCGCGGCCGGCAGCGCTCACGGGGTAGGAGTAGGCGCCGATGTCCT

CCACGCTCAGGGGACGCCACTGGCCCCTCATGTCCTCCCCGGGGAGCCGG

GCGGTCCCCGGCTTGGCCCGCAGGCTCCACAGGTTTGGGGGCATCAGGGC

CTGCTGGGGACCCGGAGAGGCCGGGAAGCGGAAGGTCTCGGCCGGGTACG

GTGACATGGTCCGCCCGAAGCCTGGGGCCACTTCGGCCTCCAGCGGGGCC

GCCACCGCGGCCGTGGCCTTGGCAAAGCGAGGGCTGCCCTCGTAGGTGGT

GGCGGGTGGCTTGCGGTCGAAGAGCTCGTCGCGGCTGTTCAGGTAGATGG

CCTGCTGCGACGCGGTCAGCGTGCTGGCCTGCGCGTGCTCCTTCTCCTCC

GACGTGGCGCTGAAGCTGGAGTAGGAGCTGGACGTGGGCAGTGAGCCTGC

GTAGCTGGGGAAGGCCTCATGCTGGAAGCCCGCCGGGAAGGCCGCCGCCT

CGGCCTCCTCCTCCTCCTCGGCCGCGCTGTCAGTGGAGTTCTGGGCCCGC

AGGAAGCCCACGTCGGTCACGGGCGCGTCCACGCTAGGCCGCCGGTCTCG

CCGCCGCTCCTCCGGGCAGTAGAGGGCTGTGTCACTGCAGTAGATGTCTC

CCTTGTAGGGGGGCCGCGGGCCTGGTTTCTCCACCCCGTCGCAGAAGGCC

AGGTCGCGGGCGGAGGCATCGGACAGGCGGGAGGACAGGCTGGCGGGGTC

CGGCTTCTCCAGCACCTTGGCAATGACGCAGGTGGGGACGCTGTCGGCGT

AGGCCGGGTGGCAGAGCGGGGATGGCAGGCTGCAGCCGTGCTTCTCCATG

TGCAGGCTCACGCGCTCCTGGAAATCCGAGGGCAGCTGGAACGGGTGCGA

GGAGGGGGACGGCG

>hg19_cpgIslandExt_**CpG: 36** range=chr14:101349373-101349860 5'pad=0 3'pad=0 strand=+ repeatMasking=none

CGGATCCGTCTGAGCTTGGCTGGTCGGAAGTCTCATCATCTGCTTCCTTC

GGGTTAAACACGTCGGCCAGGTCTGAGTATGGGTGTGGCAGTCCGGGTAG

CAGGCTCATGCCGTGCCTCTCTAGGGCAATGCATGGCGGGGGCGGGCGGA

AGCAGTTCTTCAGGCAGTAGGGAGAGTGGAAGGTGCAGCGGCCTTTGATC

CAGTCGACTTCGGGGGCGTGGACTCGGAGCCAGCGGATGCCTAGGACCAC

AGAGAAGTTCGGTGAAGGTACGATGTCAAATTCGATGGACTCCTGGTGGT

TCTGGTGGATACACACCAGGGGCTCCGTGTAGAGCCAGACAGGCTCGTTG

CCAATCAGCGAGCCGTCCACGGATTGGACCGGCTGTGGGTACGGCTTCTC

GTAGAGCTCGACGTAGTGCTCTTGGGCGAACTTCTCATCCATGAAGTTGC

CGTCAGCTCCCGAATCCACCAGGGCCTGGACCGCGACG

>hg19_cpgIslandExt_**CpG: 48** range=chr14:101531644-101532384 5'pad=0 3'pad=0 strand=+ repeatMasking=none

CGGGGAGAGGTTACCCGAGCAACTTTGCATCTGGACGACGAATGTTGCTC

GGTGAACCCCTTTTCGGTATCAAATTCCACCAGGGAGGCCGTCTTGGAGG

CTGGGGCACCTCGGGGAAGGACGCCGGCATCAGCACCATTCTGGGGTACG

GGGATGGATGGTCGACCAGTTGGAAAGTAATTGTTTCTAATGTACTTCAC

CTGGTCCACTAGCCGTCCGTATCCGCTGCAGCCTGTGGGGCCTGCGGGCC

GGGGAGCCGATCGCGCTTCAGCTCAGCGCCTTTCCTGGTACTTGAAGGGA

GATCGACCGTGTTATATTCGCTTTATTGACTTCGAATAATACATGGTTGA

TCTTTTCTCAGTATCAAATCTCACCTTGGAGGACCCGTTGGAGATGAAGC

CCTTTTGAGGGTAGGAGCAGGACGGGTGCTTTGGAGTCGCTGCCTCAGTG

GGGCCTGGTCACCGAGAGTGGGCCGGGCAGGGGTCTCTTTCCTGTCTCTG

TGTCTTCTCACCTTTGATGTCCCATCCGTCCTCAGGACTGCTTCCCGGGG

GCAGCGCTGGCACCACGGGACGCGGCAGCCACGTTCTTGAGCCGATGGCA

CTCTGGGTACCTGAGAAGAGGTTGTCTGTGATGAGTTCGCTTTTATTAAT

GACGAATATAACACAGATGGCCTGTTTTCAGTACCGCTACCGCCCGGTGG

TGTGCGGGCGCCACGCCTGAGGCGGGACTTTCCAGGGTACG

>hg19_cpgIslandExt_**CpG: 407** range=chr14:102025990-102031567 5'pad=0 3'pad=0 strand=+ repeatMasking=none

CGAAAACCGGAGGGGGGGACCTCCGCCCAAGGCAGGCGTCTCTGCGCGCC

CGCTGGGGCCCTGGTCCTGCAGTTGGCTTCTCCCGACACGTTCCTTCGCT

GCCGAAAGCGATTCGCCCGCGCTCTACCGAGCCCAGCCAGCTCCTACCTC

GGCCCGGGCGGCGAGCGCTGTCTCCGTCCCGAGAAGAAGTTTCGCGGCTG

GTCCCGGGGCGGCAGGCGCACGGGACGCTGGCGGCGGCGGCGGCTTCCGA

CTTGGGCTCAGACCCCGCCGCTCCAGACAAGTCCCCGCGACTGCGACAGT

GTCTCGGGCAGTTCAGCTCCCCAGACGGGAGACAGACCCTCGCGACCTGG

TTCAGGCCTTGCCCTGGGGGACACTGCAGTCCAAGGCGAAAGGAGACTTG

CTCCCGGCCTCTGGGTCCCTAGGTCAAGGCACCTCCCCGCCCCGCCGCCC

CTGAGCCCAAGTCGCCAACCCCGCGCCGCGCGCAGCGAGGGATCAGAAGG

TCTCGGACGCCGCGGCAGGGCCGGCCAGGGCCCCACGAGTTAGGGAGTTG

CCCCGCACCCCTAAACGCTCAGCACCCATTTACGGTTGAAAGTACGCACT

TGACGCGTCCGGGCGCCTCCGCGGTGAGTCGCGCCGCCGCTCTCGGGGTC

CGCGAAGGTGGCTCAGTTCGGGCGCTCCAGTCTCGACGTTCCCGGGGTAG

AGAGCAACGTCCGGGGACGAACGGGACGGGTGCGCCCAGCGGCCGGGGCT

GCGCTGGGCGAGGCAAGCGGCGAGAGGGGAGCGCTCCGGCGTTCAGGCAA

CAGCTACCCCTAGTGGGAGAGCCGCCCGGGGCCGGCGCCGCCCCGCGCCC

GCTGCCCCGCGCCCCACGCCCGTGGCGCCCCGCGCCCGGGTCGGCTGGGG

GCGCAGGGCCGGCGCCGCTTTCCCCAGCTCTCCGGTCTCCGGCGCCCGGC

GGTAACCCGCCCGCGGGCTCTGCCCTCCGCACCTCTCAGCCCGGCCCCGG

CGGCCGCTGACCCAGGAACCGCGGCGCCCCGCTGGAACGAGATGGGACGG

GGCGGGCGCGGCCAGGGCGGCGCGGGCTGGGGCGGCGCGCGGCAGCCTCG

GGCAGCGGCGGGGGGCGCGGAGCGCGGTGTGCGTGGCGGGGCGGTGCGGG

GGGCGGCCGTGTGCGAGGCGCGAGTGTGAGCGCGCGCGGGAGGCGGGCGG

GCGGGCTCCGGCAGGCGAGCGGCGCCCGCGGGCGAGCCAGGAAGGCGGCG

GGAACGCAAAGTTGCCTCCTCGCTGGCCCGCGTCCTCGGTGGGGCGGGAG

CCGGGGCCACCGAAGCGGCGGCCGCGGACCCGGCCTCCCGCGGCACCTGG

GCAGCGGCCCCGCACGAGCGGCAGCGGCGTGGGCGGCGTTGGCGGCGGCG

CGCGGGAAGCGAACCGGAGCTCCGGCGCGGCTCGGCGGCCGCCGGGGAGC

TCGGCTCAGGTGCGCGGCGAGGGGGGCGCGGGCCGGAGCCAGGCGGCGGG

GACCAGGGCGGCGCGCAGCGCTCGCGCCAGCTGGAGGACCTCCCCAGCGG

ACGCCCAGGTCCCTGGTCGGGCTCCCGGTCCCCAGCGGCAGCGGCTGCCG

GAGTCCCCCGCCCCCGAGAGCTGGCTGCGGGGCCCGGGCCCGCCCCCACC

GGCCCCAGGCCCGGCCGCTCCGCCCTCCGCCCGCCTGCGCCCGCCCTCAG

CCCAAGATTTCTAGGGCATTGGCCGCGCTGCTGGGTGATCCCTCCGGGCT

CAAGTTGCAAGGGGGCGGGCCGGGCCGGAGGTGGAGTCTCCCGCCAATTG

AAGCCTCCGCTATAAATTGAACTCCCTGCACTGCTGAAGCCCAGATGCCT

CGCCAGGCCACGTCGCGGTTGGTGGTCGGAGAGGGCGAGGGGTCCCAGGG

GGCTTCGGGGCCTGCAGCCACCATGCTCCGCTCCCTGCTGCTTCACTCCT

TGAGGCTCTGCGCCCAGACCGCCTCGTGCCTCGTGCTCTTCCCGCGCTTC

CTCGGCACGGCCTTCATGCTCTGGCTTCTCGATTTCTTGTGTATCCGCAA

GCATTTCCTGGGCCGCCGCCGCCGGGGGCAGCCCGAGCCCGAAGTGGAGC

TCAACAGTGAAGGCGAGGAGGTGCCTCCCGATGACCCGCCCATCTGCGTG

TCCGACGACAACCGCCTGTGCACCCTGGCGTCGCTCAAGGCGGTGTGGCA

TGGCCAGAAGTTGGATTTCTTCAAGCAGGCGCACGAGGGCGGTCCGGCGC

CCAACTCCGAGGTGGTTCTGCCCGACGGCTTCCAGAGCCAGCACATCCTC

GACTACGCGCAAGGGAACCGCCCGCTGGTTCTCAATTTCGGCAGCTGCAC

CTGACCACCGTTCATGGCGCGCATGAGCGCCTTCCAGCGCCTGGTCACTA

AGTACCAGCGCGACGTCGACTTCCTCATCATCTACATCGAGGAAGCGCAC

CCCTCCGACGGCTGGGTCACCACGGACTCTCCCTACATCATCCCACAGCA

CCGGAGCCTGGAGGACCGGGTCAGCGCAGCGAGGGTACTGCAGCAAGGTG

CACCCGGCTGCGCTCTGGTCCTCGACACCATGGCCAACTCCAGCAGCTCG

GCCTATGGCGCCTACTTCGAGCGTCTCTATGTCATCCAGAGTGGCACTAT

TATGTACCAGGGCGGCCGTGGCCCCGACGGCTACCAGGTCTCTGAGCTGC

GCACTTGGTTGGAACGCTATGATGAGCAACTGCACGGCGCTCGGCCCCGG

AGGGTGTAAACATCCAACGGACAATTGACTGAACTTGGTGGGCTGGGCCT

TCGAGCCTTCGAAGCCCACGTGCAAGCGCCTCAAACCAAGTCACGCTTGG

CGAGGCCCCAGTGACACTGATGTGCTGAGCCACCATTTCAGACTGAGTCT

GCACCCTCAGCCACATGAACAATCTCCCCTACCTCCCTGGGACTCTGCTT

CTGTAACTGTCTCATTCACACCTGCCTGGCTCACTGGAAATCCTCTTTTG

AGCGCGGGATATGGCTTGCCCTTGTCCGTGTGCCCCCAGGACTTTGCCTC

TACAGCATTTTCTTACACCCCCTCCCCAGCGTGCCCTCAGCCAAGTGCTT

TGGCCCGGTGCTTCCCGCAGCTGCACAGAGACCTTGGCCACGCCCGCGCG

CCCTGAGCGCAGCTGGGTTCCAGGAGACTCTCAGCTCAGCTGAGCTAGTT

GCCTGGCACCCACCTGTCGCGCGCGGAGAGGGGGTTCCCTGTTGCTTTTG

TGTCTGTTTCCTGTCCCTGGTAGGGGAAGTGATGTCGTGGATGGGGAGGG

GTGGGCAGGGTAGTTTCCCCCGCTTGTTTTGGGTGCACAGGAGCCCCACT

GCTGATGACGAACTATCTCTAACTGGTCTTGACCACGAGCTAGTTCTGAA

TTGCAGGGGCCTCAAAGCAGCACCTAAACCTTGAGGGGGAGGGTGCTCTG

GGTTTCCGTGAGGTAACCACCTTAAATGGGAGGGAAGTTGGGGTGTCTGC

TTTGGGACCAGAGGAAGATAGCTTGAGAGGCATTGGCGAGGTTCGCAGCG

CCCCAGGGAGAGAGAAAAAGCTGAGACTCCTGGGGAATGACGTTGGGGTG

ATGGAGTCCGGGGAAAGAGAGGTGGGGGGAGAGCCTGAGGTCCCCAAGTG

AGGGGAGTCCTAGGCAGAGCTGCTGATTGTGGGGCTGGGAGGTGGAGGGC

CCCTGATTCGAAGGCCATTTGGTGAGTGTTTTGCTGGAAATATTTCCTGT

ATATAAACTTCTTTCAATCTACAATAATAAAGGCTTGAGGTAAACTGCTT

TCTGGTTGCCTTTGGTCCCTTTATTTTCTTTTCCCTCTGAATAGCGGCAG

GAATTAAACACAAATCCCTCCCTCGCGTCCCCATAGTGACCTTGGGAAGC

GGGACGGGAAGGAGCCTAGGCTGGCAGGGGCGCTGGAGGAGGATGGAGGA

GGAAGAGGGTCTGCGGCGGTGCCCGGGGCGCTCCTACAACCGCACGCACC

CACCTGGACGGGGAACTTAGCTGCGGGAGAAGAGCGAGGTAGCGGAGCGC

CGCGGGATCCTGGAAAGCGGCGGGAGAGGCAAGGGGGCCCTCTCTTGCTC

CCGCGCGCGCACACACAGACCGAACAGATGCAGCTGCCCCTGCAGCCGCC

GATCCAACTTAACTGAGTATCCGGCGGTGTGTGGAGGGGGTTGGGTGAGG

TGGAGGGCGAGAGGGGCTTTGGACCCGGTACAGCCTGGCTCCCGACCGCT

GCAGGCACCACTGCGCACCTGACTCCTCCCTGGCCCCCTTCCGGCACCTG

CAACCTTGCCTCTTGCCCACCCGCTCCAGGCAGCTGAGGTCCGCTGGAGG

CGCGCCTTGGCAGAGGGGCGCCCTCACTCCAGCTGCGGCTCCTACTGCCG

GGCCAGCGTGCTGCTGGGTGCGCCCCCTGCCCAGGACCCCCCTCCTCCTG

TCCCTCCTCCGCTTCCCCAAGCTCCAGTCAAACCCCACCACCCGCTCCGT

GCTGGGAAGCAGAGGTGGGAGGCCGCTGGGATGAAGAGAGAGAGACCAGG

GAGAGGTGAATGGTGGATTCCAGGGCTGGGGCGGGGATTCGGGGCCTTAG

GCACAGGTCCCCTCTTGCAGTCACGGCCAGGGGCTCGCTCTCGCGCAATC

CCCGCCCTCACTGTCTCGCCTGTTGCGCCCGGAGTGAGGGCGACCCGGGG

GACGCGGGATTAGCTTAGCCCGCAGCCCCTGTGCCAGCGAAACCTTGAAC

ATTAAGTGAAAGGTGGGGGACACAGTGAAAGCCGCTGAGGCGGGAGTGTA

CGGATCCAGGTGAGGGGACTGGGCACGGCAACTTCCCTGGCGCGCCAGGA

CAATGCCTGGGAACCCAGGATTGTCAGGGTCGCCGGGGGTGGGGGTGAGT

AGTGTCGGTTTGCGCGCTTGGATGTGAGCGCGGGTGTACATGGCAGGGTT

GGGTAGGGGTGCAGAGGGGCGCGGCAGGCAGGATGGAAGAGACGCCCCCG

TTCAGCCAACGGCGTCCCTAATTCTGCGCCCAGCCCCCGCGCTGCTCCTC

CGTAAAAACTTTTTGAAACTGACATCCTAAATTTGTGGATTTTTTTTTCC

TTTAAAAGTATCTCAATTGAAAAATAACTTTAAAGAAAACTTTGGCACCC

ACGAAATTTCTGCATCTCCTCCCCACTACCACCAACCCCTACCTACCTCC

GCGAACGCTCCTTCTCATCCCGAAACCGCCAGGAAGCAAAGGAGATCGCG

GCAGGGACGCGGCCCGGCGCCTGGGAGAGTCCTCCCTGACCTCCCCGCAC

GCGCGGCTGGCCGGGACTGGAGCTTGGTGGCGCGGAGGGTGGCCTGGGAC

AGCGTCAGCCTCCCGGCCCAGCAGAGACCGCTCCAGCCGCTCCGCGTCCC

TACCGGTGGCTTGGGGGTCCCCTTGGACGCGCCAAGCGACCTCTCCTACC

CGGAAAGCCTGCGCCCCCTGGGCTTCCTGGAGCTTCCCAGATCGAAACTC

CAAAAAGCGCTTTCGCGCCGCAGCGCCGGGACCTGCAGCGCCAGCCACTC

CCCGCACGGGCCGCACTCGCTCTGGTCG

**Immunohistochemistry (IHC)**

Serial 4 μm thick sections were processed for haematoxylin and eosin staining and for immunohistochemistry. Endogenous peroxidases inhibition was obtained by incubating slides in 3% H_2_O_2_–methanol for 20 min at 4°C. Antigen retrieval was achieved by boiling slides in citrate buffer (pH 6.0) using a microwave oven. Immunoreactivity was revealed with the EnVision system (Dako) and Diaminobenzidine (DAB) as chromogen (Sigma-Aldrich). Counterstaining was obtained with Meyer’s hematoxylin. Positive staining was quantified by using Image J software (NIH) on 6 randomly selected consecutive fields at a 20X magnification.

**Supplementary Figure Legends**

**Figure S1.** Study flow chart.

**Figure S2.** (**A**) Correlation graph between miR-494 and miR-495 expression in surrounding liver cirrhosis (LC) from HCC patients. Axes report the 2^-ΔΔCt^ values corresponding to miR-494 or miR-495 levels transformed in a log2 form. (**B**) Correlation graph between *PROM1* and *EPCAM* mRNA levels in HCC and (**C**) surrounding liver cirrhosis (LC) from HCC patients (N=28). Axes report the 2^-ΔΔCt^ values corresponding to mRNA levels. All values are transformed in a log2 form. (**D**) Correlation graph between miR-494 and *ABCG2* mRNA levels in HCC rat samples (N=23). Axes report the 2^-ΔΔCt^ values corresponding to miR-494 or *ABCG2* levels transformed in a log2 form. (**E**) Western blot analysis of *PROM1* and *EPCAM* expression in human HCC samples. B-actin has been used as housekeeping gene. Correlation graphs between miR-494 and *PROM1* or *EPCAM* expression in HCC human samples (N=18). Y-axis reports the 2^-ΔΔCt^ values corresponding to miRNA and protein levels transformed in log2 form. (**F**) Western blot and QPCR analyses of AFP expression in rat HCC samples. B-actin has been used as housekeeping gene. Correlation graphs between mRNA and protein AFP levels and between miRNA and AFP protein levels in rat HCC samples (N=11). Axes report the 2^-ΔΔCt^ values corresponding to protein, mRNA and miRNA levels transformed in a log2 form. (**G**) QPCR analysis of miRNA expression in miR-494 stably overexpressing Huh-7 cells. Y-axis reports the 2^-ΔΔCt^ values in a linear scale. U6RNA was used as housekeeping gene. PMXs: empty vector infected control cells; miR-494: pMXs-miR-494 overexpressing vector bearing cells. (**H**) Western blot analysis of *PROM1* expression in tumor masses derived from miR-494 stably overexpressing and control vector (pMXs) Huh-7 cells. B-actin has been used as housekeeping gene. (**I**) Image of the DLK1-DIO3 locus and relative GpC islands (green bars and boxes) as displayed by UCSC database (http://www.genome.ucsc.edu/). Tested CpG islands are underlined with a red line. Principal genes as well as snoRNAs and miRNAs cluster are mentioned in the figure. (**J**) Correlation graph between primary and mature miR-494 expression levels in HCC patients (N=39) and (**K**) HCC cell lines (N=8). Axes report 2^-ΔΔCt^ values transformed in a log2 form. (**L**) Complementary miR-494 binding sites in *DNMT3B* and *DNMT3A* 3’UTRs, as displayed by MiRanda algorithm. Stars represent bases mutated in 3’UTR vectors used in the luciferase-reporter assay.

**Figure S3.** (**A**) QPCR analysis of miR-494 expression in HCC-derived cell lines. Y-axis reports 2^-ΔΔCt^ values in a linear scale. U6RNA was used as housekeeping gene. (**B**) Complementary miR-494 binding sites in *CDKN1B*, *PTEN* and *BBC3* 3’UTRs, as displayed by MiRanda algorithm. Stars represent bases mutated in 3’UTR vectors used in the luciferase-reporter assay. (**C**) WB and QPCR analyses of target genes in miR-494 stably overexpressing Huh-7 cells. PMXs: empty control vector. β-actin was used to normalize QPCR and WB data. Numbers represents the fold change in protein or mRNA levels in miR-494 overexpressing cells with respect to control cells. (**D**) Luciferase reporter assay in HepG2 cells co-transfected with pGL3-3UTR mutated vectors and miR-494 or negative control (NC). (**E**) Column chart reporting cell populations (percentage value) in different phases of cell cycle as detected by FACS analysis in miR-494 stably overexpressing and control (pMXs) cells. Columns and bars represent average ± SD values from three independent experiments. (**F**) Six-well plate image of colony forming unit assay and quantification of colony number are shown. All colonies were counted independently from their dimension. Columns and bars represent average ± SD values of 3 wells from two independent experiments. Initial number of seeded cells: 500/well. Days of cell growth: nine.

**Figure S4.** (**A**) Western blot analysis, cell viability and caspase 3/7 activity assays in miR-494 stably overexpressing Huh-7 cells in basal or (**B**) hypoxic conditions. (**C**) QPCR and Western blot analyses of HIF1A expression in miR-494 transfected Huh-7 cells in basal conditions. NC: pre-miR negative control; pMXs: empty vector. β-actin was used to normalize QPCR and WB data. *, p<0.05.

**Figure S5.** (**A**) Cell viability assay, caspase 3/7 activity assay and Western blot analysis in miR-494 stably overexpressing Huh-7 following sorafenib treatment. (**B**) QPCR analysis of miR-494 in stably overexpressing Huh-7 following anti-miR-494 (AM) transfection. (**C**) Caspase3/7 activity assay in stably overexpressing Huh-7 cells following AM-494 transfection and sorafenib treatment. NCi: anti-miR negative control; pMXs: empty vector. β-actin was used to normalize WB data. *, p<0.05; **, p<0.01.
