# Supplementary material for: The epigenetically regulated miR-494 associates with stem-cell phenotype and induces sorafenib resistance in hepatocellular carcinoma
Source: Cell Death Dis. 2018 Jan 5;9(1):4. doi: 10.1038/s41419-017-0076-6 (PMC5849044; doi:10.1038/s41419-017-0076-6)
Supplement: Supplementary file 2 — Supplementary Tables [file 41419_2017_76_MOESM2_ESM.doc]

### Supplementary Tables

**Supplementary Table S1**. Patient characteristics, whole (N=75) and sub-cohort (N=38)

| **Factors** |  | **Frequency(%)**  **75 pts** | **Frequency(%)**  **38 pts** |
| --- | --- | --- | --- |
| Etiology | HBV | 15/75 (20%) | 8/38 (21.0%) |
|  | HCV | 50/75 (66.7%) | 22/38 (57.9%) |
|  | Alcohol abuse | 3/75 (4%) | 2/38 (5.3%) |
|  | None | 7/75 (9.3%) | 6/38 (15.8%) |
| Cirrhosis |  | 72/75 (96%) | 34/38 (89.5%) |
| Serum AFP | > 20 ng/ml | 41/75 (54.7%) | 15/38 (39.5%) |
| Edmondson grade | I | 2/75 (2.7%) | 1/38 (2.6%) |
|  | II | 12/75 (16%) | 11/38 (28.9%) |
|  | III | 54/75 (72%) | 23/38 (60.5%) |
|  | IV | 7/75 (9.3%) | 3/38 (7.9%) |
| Barcelona Clinic Liver Cancer stage | A | 65/75 (86.7%) | 29/38 (76.3%) |
|  | B | 10/75 (13.3%) | 9/38 (23.7%) |
|  | C | 0% | 0% |
|  | D | 0% | 0% |

**Supplementary Table S2.** Primer sequences and PCR conditions for cloning experiments.

| **Cloned Portion** | **RT-PCR Primer Sequences** | **PCR product (bp)** | **Melting T (°C)** | **Cycles** |
| --- | --- | --- | --- | --- |
| *MIR-494* | Fw 5’-CGA**CTCGAG**ATTCGGGGAATCTTCCTGGAG-3’  Rv 5’-TCGA**CTCGAG**GCACGCTGTCAGCCTCGC-3’ | 299 | 62.5 | 35 |
| *P27*-3’UTR | Fw 5’-TAC**TCTAGA**CAAAAGAGCCAACA-3’  Rv 5’-TGCT**TCTAGA**GGATGTCCATTCC-3’ | 245 | 59.0 | 35 |
| *PTEN*-3’UTR | Fw 5’-TGATA**TCTAGA**GTATTGATTGATTGCTC-3’  Rv 5’-ATT**TCTAGA**ACTAAACATTAAACTTGTACC-3’ | 878 | 63.1 | 30 |
| *PUMA*-3’UTR | Fw 5’-AG**TCTAGA**AGTGGGGAGGG-3’  Rv 5’-TCCACTGT**TCTAGA**CTGATTTTATTGA-3’ | 155 | 62.4 | 40 |
| *DNMT3B*-3’UTR | Fw 5’-TGTT**TCTAGA**CGTGTGCAGTTGT-3’  Rv 5’-TTA**TCTAG**ACATAAGGTAAACTCTAGACATC-3’ | 990 | 60.0 | 36 |
| *P27*-3’UTR-mut | Fw 5’-AAACAGCTCGAATTAAGAATATGTT**AG**CTTGTTTATCAGATACATCACTCT-3’  Rv 5’-AGCAGTGATGTATCTGATAAACAAG**CT**AACATATTCTAATTCGAGCTGTTT-3’ | - | 78.0 | 18 |
| *PUMA*-3’UTR-mut | Fw 5’-GTTTGGTTAATTTTTTTTGTACATGATTTTTGTATGT**GG**CCTTTTCAATAA -3’  Rv 5’-TTATTGAAAAGG**CC**ACATACAAAAATCATGTACAAAAAAAATTAACCAAAC-3’ | - | 78.0 | 18 |
| *DNMT3B*-3’UTR-mut1 | Fw 5’-CTGTGCCTTG**AGA**CAACAGTTTTTGCTAATTTTTAGGCTGAAAGATGACGG -3’  Rv 5’-GTCATCTTTCAGCCTAAAAATTAGCAAAAACTGTTG**TCT**CAAGGCACA -3’ | - | 78.0 | 18 |
| *DNMT3B*-3’UTR-mut2 | Fw 5’-TTTTAAAATCTCAAACTTCTATTTTTATG**AGA**AACGTTTTCATTAAAATTTTTTTTGTAA-3’  Rv 5’-TTACAAAAAAAATTTTAATGAAAACGTT**TCT**CATAAAAATAGAAGTTTGAGATTTTAAAA-3’ | - | 78.0 | 18 |

# **Supplementary Table S3.** Primer sequences and PCR conditions.

| Analyzed genes and DNA regions | **Primer Sequences** | **Species** | **Amplified product (bp)** | **Anneling T (°C)** | **Cycles (N.)** | Analysis |
| --- | --- | --- | --- | --- | --- | --- |
| Pri-miR-494 | Fw 5’-AGTGTTCTCTGTATTGGCGG-3’  Rv 5’-TTCTATTTGCGTGTCCCAGG-3’ | Human | 110 | 61.8 | 45 | qPCR |
| P27 | Fw 5’-TCTGAGGACACGCATTTGG-3’  Rv 5’-TGTTCTGTTGGCTCTTTTGTTT-3’ | Human | 138 | 62 | 45 | qPCR |
| ***PTEN*** | Fw 5’-TGGAAGGGACGAACTGGTG-3’  Rv 5’-CATAGCGCCTCTGACTGGGA-3’ | Human | 174 | 62 | 45 | qPCR |
| ***PUMA*** | Fw 5’-CGACCTCAACGCACAGTAC-3’  Rv 5’-CCTAATTGGGCTCCATCTCG-3’ | human | 146 | 62 | 45 | qPCR |
| ***ß-ACTIN*** | Fw 5’-ACCTTCTACAATGAGCTGCG-3’  Rv 5’-CCTGGATAGCAACGTACATGG-3’ | Human | 148 | 58 | 45 | qPCR |
| ***ß-ACTIN*** | Fw 5’-CAAGAGATGGCCACGGCTGCT-3’  Rv 5’-TCCTTCTGCATCCTGTCGGCA-3’ | Human | 275 | 55 | 25 | RT-PCR |
| ***AFP*** | Fw 5’-AAAGACCAGGATCAGGAAGC-3’  Rv 5’-CCATCAAACCGAAAAGCTCAC-3’ | Human | 150 | 61.8 | 45 | qPCR |
| ***PROM1*** | Fw 5’-GTGGATGCAGAACTTGACAAC-3’  Rv 5’-ACCCTTTTGATACCTGCTACG-3’ | Human | 142 | 61.8 | 45 | qPCR |
| ***EPCAM*** | Fw 5’-CAATGCAGGGTCTAAAAGCTG-3’  Rv 5’-CACCCATCTCCTTTATCTCAGC-3’ | Human | 140 | 61.8 | 45 | qPCR |
| ***NANOG*** | Fw 5’-GAAATACCTCAGCCTCCAGC-3’  Rv 5’-GCGTCACACCATTGCTATTC-3’ | Human | 148 | 61.8 | 45 | qPCR |
| ***OCT4*** | Fw 5’-GTGAAGCTGGAGAAGGAGAAG-3’  Rv 5’-TCGGCCTGTGTATATCCCA-3’ | Human | 133 | 61.8 | 45 | qPCR |
| ***SOX2*** | Fw 5’-CACACTGCCCCTCTCAC-3’  Rv 5’-TCCATGCTGTTTCTTACTCTCC-3’ | Human | 118 | 61.8 | 45 | qPCR |
| ***NESTIN*** | Fw 5’-TGCGGGCTACTGAAAAGTTC-3’  Rv 5’-GGCTGAGGGACATCTTGAG-3’ | Human | 128 | 61.8 | 45 | qPCR |
| ***CD90*** | Fw 5’-GAGATCCCAGAACCATGAACC-3’  Rv 5’-TGCTGGTATTCTCATGGCG-3’ | Human | 146 | 61.8 | 45 | qPCR |
| ***ABCG2*** | Fw 5’-CAGGGTCATTCAAGAGTTAGGTC-3’  Rv 5’-AGAACAAGATGGAAGGATCAGTG-3’ | Human | 139 | 61.8 | 45 | qPCR |
| ***ABCG2*** | Fw 5’-GGACTCAAGCACAGCAAATG-3’  Rv 5’-AGTTTCCCAGAAGCCAGTAAG-3’ | Rat | 143 | 61.8 | 45 | qPCR |
| ***PROM1*** | Fw 5’-AGCCAAAGTATTCCTGTCGAG-3’  Rv 5’-AAGCAGACTATTAAGCCACCC-3’ | Rat | 139 | 61.8 | 45 | qPCR |
| ***EPCAM*** | Fw 5’-GCTGAGATAAAGGAGATGGGTG-3’  Rv 5’-AGGTCTATCCGTTTGCAACC-3’ | Rat | 129 | 61.8 | 45 | qPCR |
| ***AFP*** | Fw 5’-AAAGACCAGGATCAGGAAGC-3’  Rv 5’-CCATCAAACCGAAAAGCTCAC-3’ | Rat | 150 | 61.8 | 45 | qPCR |
| ***HDAC1*** | Fw 5’-GAGATGACCAAGTACCACAGC-3’  Rv 5’-TGACAGAACTCAAACAGGCC-3’ | Human | 145 | 61.8 | 45 | qPCR |
| ***HDAC2*** | Fw 5’-CATGGCGTACAGTCAAGGAG-3’  Rv 5’-ATGGGTCATGCGGATTCTATG-3’ | Human | 120 | 61.8 | 45 | qPCR |
| ***HDAC3*** | Fw 5’-GGACTTCTACCAACCCACG-3’  Rv 5’-CAGCACGAGTAGAGGGATATTG-3’ | Human | 147 | 61.8 | 45 | qPCR |
| ***HDAC4*** | Fw 5’-ACAAGGAGAAGGGCAAAGAG-3’  Rv 5’-GCGTTTTCCCGTACCAGTAG-3’ | Human | 149 | 61.8 | 45 | qPCR |
| ***DNMT3A*** | Fw 5’-ATGGGCGTTAGTGACAAGAG-3’  Rv 5’-TCACAGTGGATGCCAACG-3’ | Human | 135 | 58 | 45 | qPCR |
| ***DNMT3B*** | Fw 5’-AAGCCCATGTTGGAGTGG-3’  Rv 5’-GTCTTGTTCTCGTATTTCCTTGATTC-3’ | Human | 148 | 58 | 45 | qPCR |

# **Supplementary Table S4.** Antibodies employed in this study.

| Antibody | **Catalogue Number** | **Company** |
| --- | --- | --- |
| p27 Kip1 (D37H1) | #3688 | Cell Signaling |
| PUMA | #4976 | Cell Signaling |
| PTEN (138G6) | #9559 | Cell Signaling |
| phospho-S6 Ribosomal Protein (Ser240/244) | #2215 | Cell Signaling |
| S6 Ribosomal Protein (C-8) | #sc-74459 | Santa Cruz |
| phospo-AKT (Ser473) (D9E) XP | #4060 | Cell Signaling |
| AKT | #9272 | Cell Signaling |
| phospho-mTOR | #2971 | Cell Signaling |
| mTOR (7C10) | #2983 | Cell Signaling |
| Cleaved caspase-3 (Asp175) | #9661 | Cell Signaling |
| PARP | #9542 | Cell Signaling |
| BAX | #2772 | Cell Signaling |
| DNMT3B | #2601 | Epitomics |
| CD133 (D4W4N) | #86781 | Cell Signaling |
| EpCAM | #PA5-19832 | Invitrogen |
| SOX2 (6F1.2) | #MAB4343 | Millipore |
| OCT3/4 (H-134) | #sc-9081 | Santa Cruz |
| ABCG2 | #SAB4300689 | Sigma |
| AFP (C3) | #sc-8399 | Santa Cruz |
| β-actin (C4) | #sc-47778 | Santa Cruz |

# **Supplementary Table S5.** Primer sequences and PCR conditions of MSP.

| Analyzed DNA regions | **Primer Sequences** | **Amplified product (bp)** | **Anneling T (°C)** | **Cycles (N.)** | Analysis |
| --- | --- | --- | --- | --- | --- |
| CpG147_M | Fw 5’- AGCGAATTACGGTTAGGTTTGTAC-3’  Rv 5’- TTCCGCGAACTAATAACTCGA-3’ | 112 | 58.5 | 32 | MSP |
| **CpG36_M** | Fw 5’-TTCGTGTAGAGTTAGATAGGTTCGT-3’  Rv 5’- GACAACTTCATAAATAAAAAATTCGC-3’ | 128 | 58.5 | 32 | MSP |
| **CpG48_M** | Fw 5’- CGGTATTAAATTTTATTAGGGAGGTC-3’  Rv 5’- ACAAACTACAACGAATACGAACGA-3’ | 172 | 58.5 | 32 | MSP |
| **CpG407_M** | Fw 5’- TACGGTTTTTATGTTTTGGTTTTTC-3’  Rv 5’- ACTATTAAACTCCACTTCGAACTCG-3’ | 103 | 58.5 | 32 | MSP |
| CpG147_U | Fw 5’- GTGAATTATGGTTAGGTTTGTATGT-3’  Rv 5’- CCTTTCCACAAACTAATAACTCAAA-3’ | 112 | 58.9 | 43 | MSP |
| **CpG36_U** | Fw 5’-TTTGTGTAGAGTTAGATAGGTTTGT-3’  Rv 5’- AACAACTTCATAAATAAAAAATTCACC-3’ | 128 | 55.5 | 36 | MSP |
| **CpG48_U** | Fw 5’- TTGGTATTAAATTTTATTAGGGAGGTT-3’  Rv 5’- ACAAACTACAACAAATACAAACAA-3’ | 172 | 55.5 | 36 | MSP |
| **CpG407_U** | Fw 5’- ATGGTTTTTATGTTTTGGTTTTTTG-3’  Rv 5’- TCACTATTAAACTCCACTTCAAACTCA-3’ | 103 | 58.9 | 43 | MSP |
