# Supplementary figures and images for: The epigenetically regulated miR-494 associates with stem-cell phenotype and induces sorafenib resistance in hepatocellular carcinoma
Source: Cell Death Dis. 2018 Jan 5;9(1):4. doi: 10.1038/s41419-017-0076-6 (PMC5849044; doi:10.1038/s41419-017-0076-6)

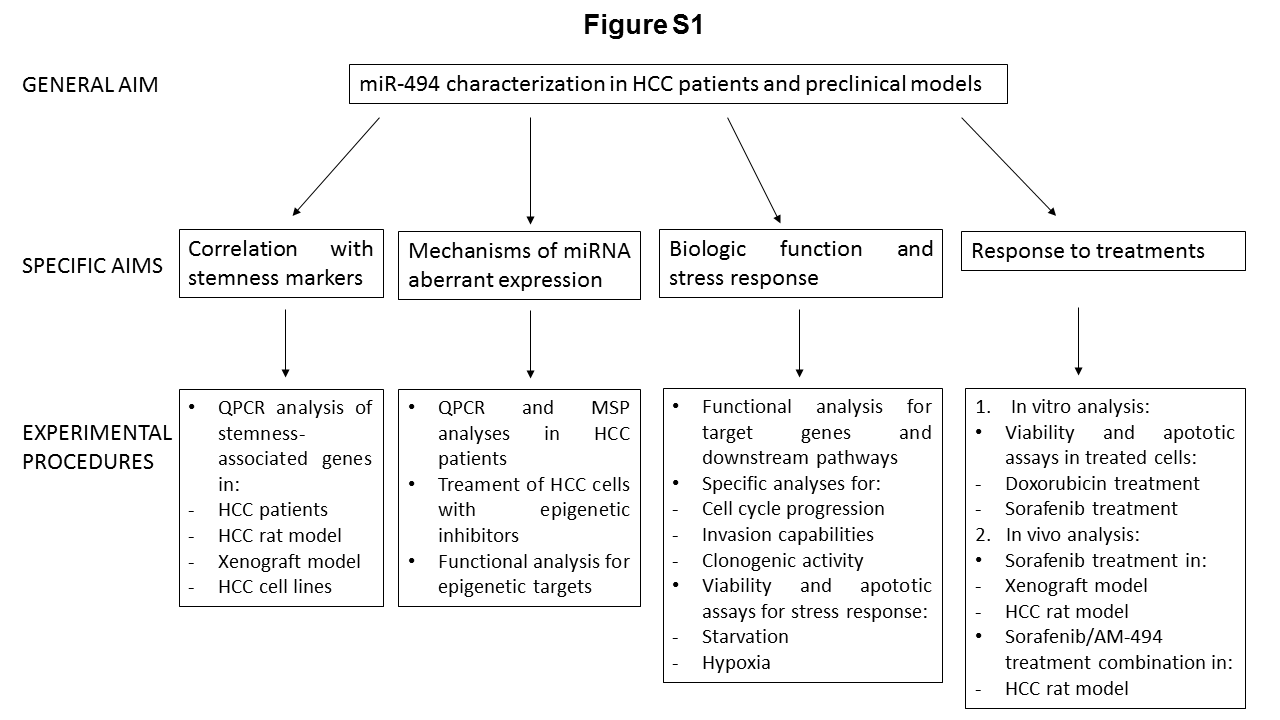

Supplement: Supplementary file 3 — Figure S1 [file 41419_2017_76_MOESM3_ESM.tif]

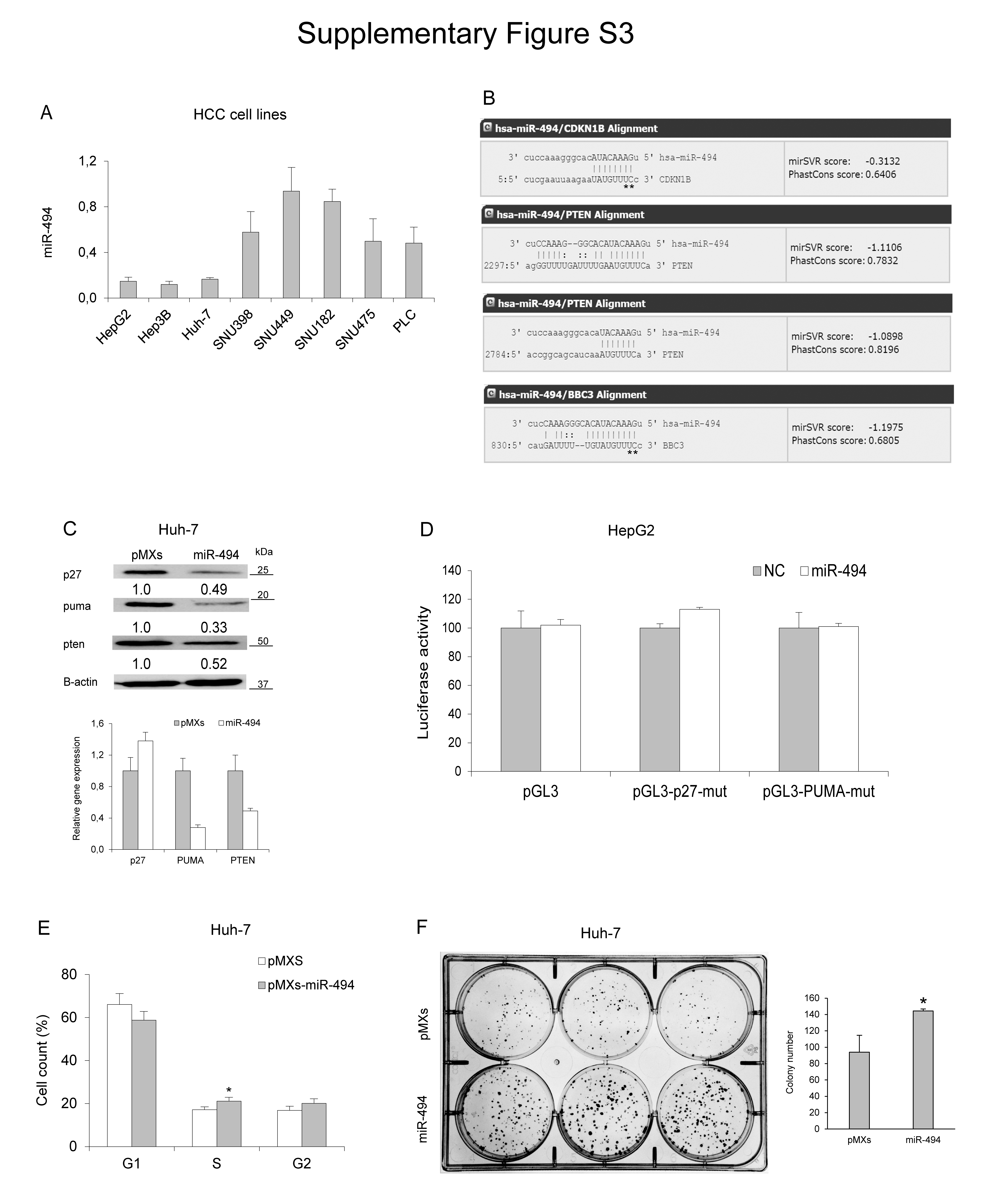

Supplement: Supplementary file 5 — Figure S3 [file 41419_2017_76_MOESM5_ESM.tif]

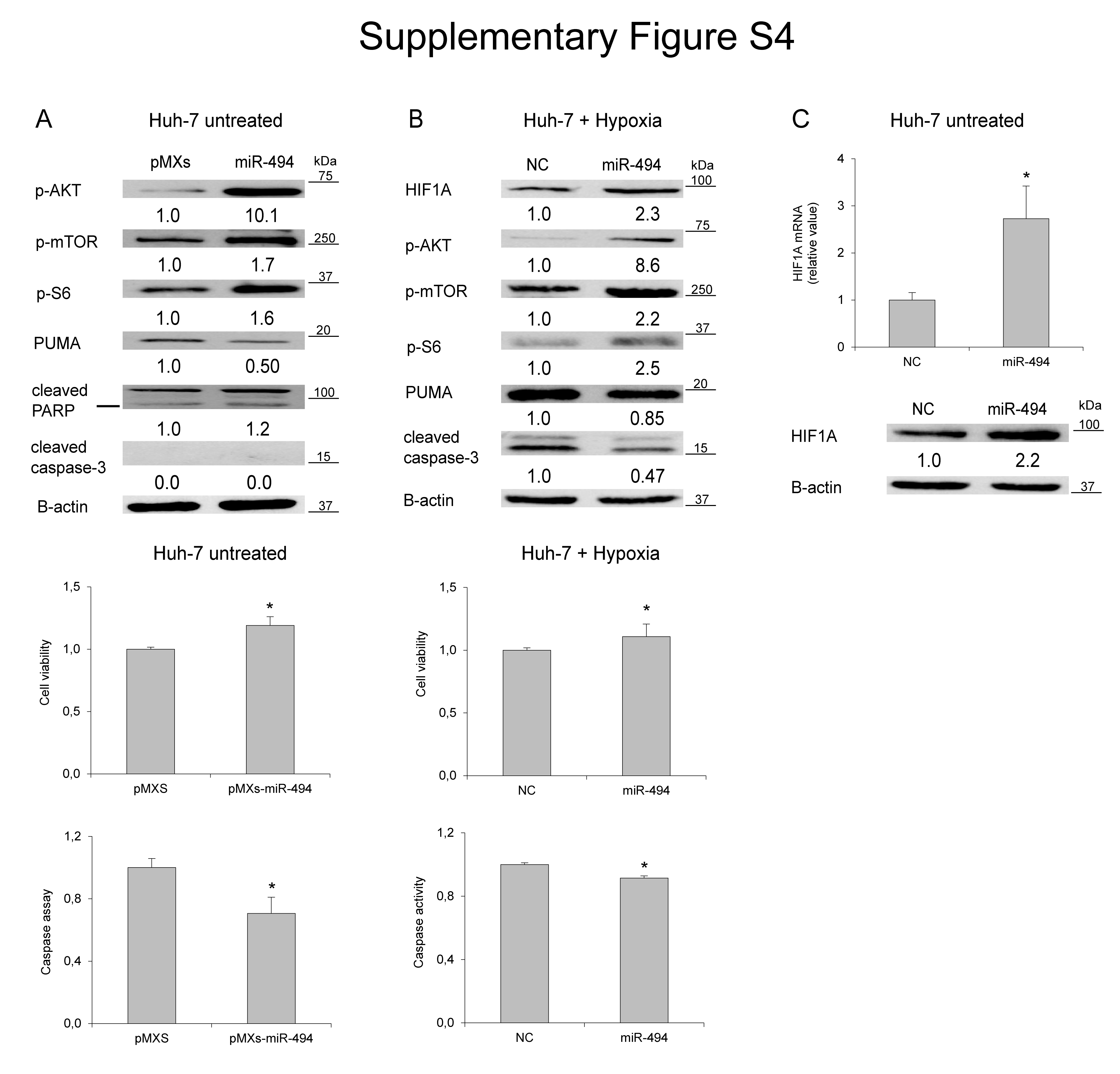

Supplement: Supplementary file 6 — Figure S4 [file 41419_2017_76_MOESM6_ESM.tif]

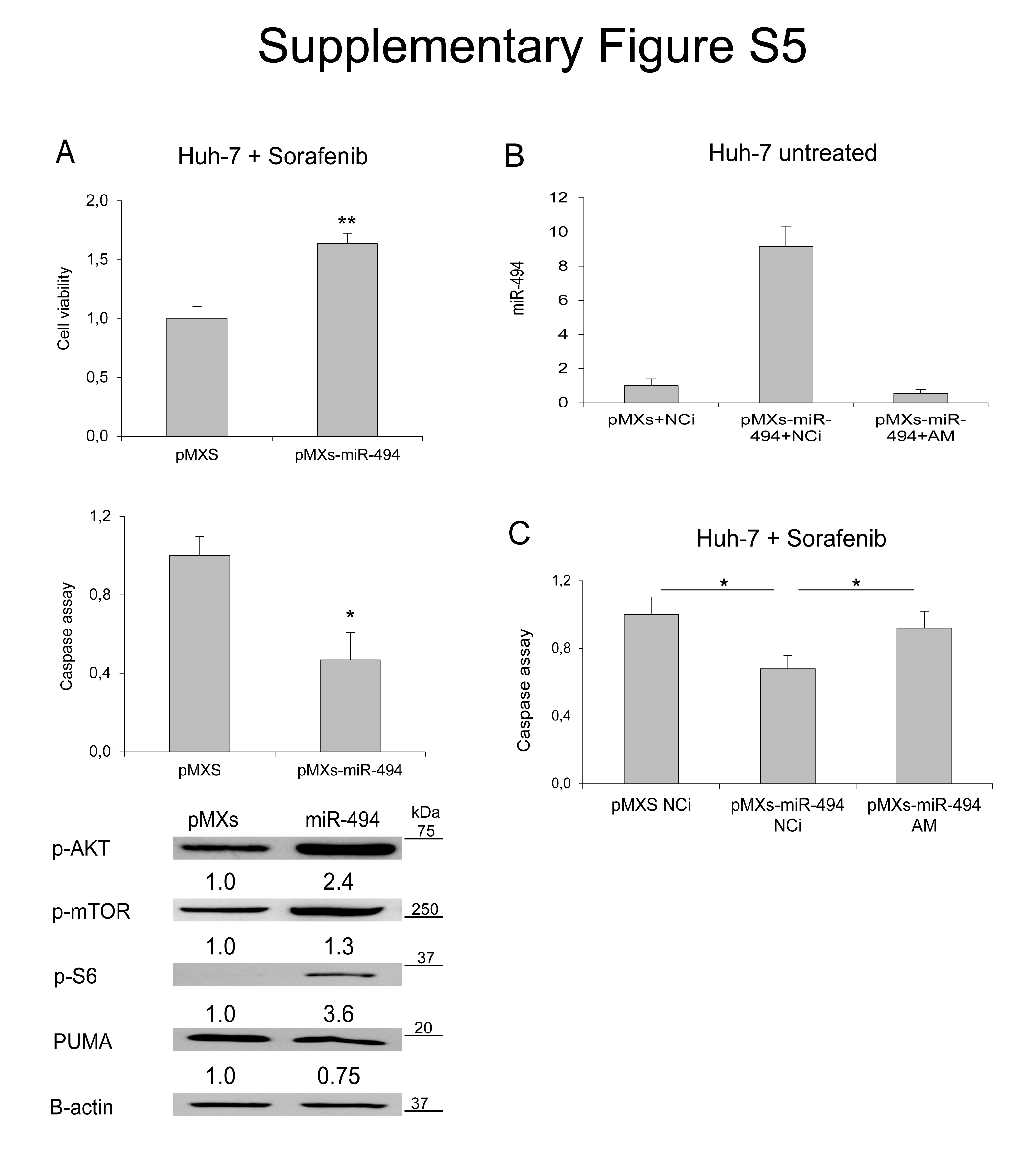

Supplement: Supplementary file 7 — Figure S5 [file 41419_2017_76_MOESM7_ESM.tif]
